# HIF-1α-mediated metabolic reprogramming in hypoxia-driven tumor metastasis

**DOI:** 10.3389/fcell.2026.1997123

**Published:** 2026-10-28

**Authors:** Shulun Shao, Mengxin Wang, Renjian Xie, Shenglan Liu, Wei Dai, Tai Zeng, Lei Lai, Kunpeng Zhang, Yumei Li

**Affiliations:** 1 School of Basic Medicine/Key Laboratory of Biomaterials and Biofabrication in Tissue Engineering of Jiangxi Province, Gannan Medical University, Ganzhou, Jiangxi, China; 2 School of Rehabilitation Medicine, Gannan Medical University, Ganzhou, Jiangxi, China; 3 School of Medical Information Engineering/Key Laboratory of Biomaterials and Biofabrication in Tissue Engineering of Jiangxi Province, Gannan Medical University, Ganzhou, Jiangxi, China; 4 Key Laboratory of Prevention and Treatment of Cardiovascular and Cerebrovascular Diseases, Ministry of Education/School of Pharmacy, Gannan Medical University, Ganzhou, China; 5 Scientific Research Center, Gannan Medical University, Ganzhou, Jiangxi, China

**Keywords:** hypoxia, hypoxia-inducible factor-1α, metabolic reprogramming, tumor metastasis, tumor microenvironment

## Abstract

Tumor metastasis is a multistep process that requires profound metabolic plasticity to support cancer cell invasion, survival in circulation, colonization of distant organs, and metastatic outgrowth. Hypoxia is a hallmark of solid tumors and a major driver of metastatic progression. As a central transcriptional regulator of the hypoxic response, hypoxia-inducible factor-1α is stabilized under oxygen deprivation and orchestrates broad metabolic reprogramming by regulating glycolysis, lactate production and export, mitochondrial respiration, lipid metabolism, amino acid utilization, redox homeostasis, and interactions with the tumor microenvironment. These metabolic adaptations not only provide bioenergetic and biosynthetic support for disseminating cancer cells but also promote epithelial-mesenchymal transition, extracellular matrix remodeling, angiogenesis, immune evasion, and premetastatic niche formation. In this review, we summarize current knowledge regarding the mechanisms by which hypoxia-inducible factor-1α-mediated metabolic reprogramming contributes to different stages of the metastatic cascade. We also discuss the therapeutic potential and limitations of targeting hypoxia-inducible factor-1α-associated metabolic vulnerabilities in metastatic cancer. A better understanding of the context-dependent functions of hypoxia-inducible factor-1α may facilitate the development of more effective anti-metastatic strategies.

## 1 Introduction

Cancer metastasis is the leading cause of cancer-related mortality and remains a major challenge in clinical management. It is a dynamic, multistep process in which tumor cells detach from the primary tumor, invade surrounding tissues, enter blood or lymphatic vessels, survive in circulation, extravasate at distant sites, and eventually form metastatic colonies (Seyfried and Huysentruyt, 2013; Castaneda et al., 2022; Chaffer and Weinberg, 2011). During this process, epithelial-mesenchymal transition (EMT) promotes tumor cell motility and invasiveness, whereas mesenchymal-epithelial transition, dormancy, and cancer stem cell-like traits support metastatic colonization and late recurrence (Phan and Croucher, 2020; Zhang Z. et al., 2025; Batlle et al., 2017). Although early-stage cancers can often be controlled by surgery, radiotherapy, or systemic therapy, disseminated tumor cells may persist in distant organs for long periods and resume growth when the microenvironment becomes permissive, ultimately leading to overt metastasis and poor clinical outcomes (Castaneda et al., 2022; Phan and Croucher, 2020).

The tumor microenvironment (TME) is a key determinant of metastatic progression. Among its defining features, hypoxia, which arises from insufficient perfusion and rapid tumor growth, imposes strong selective pressure on cancer cells and promotes aggressive phenotypes, therapeutic resistance, and metastatic dissemination (de Visser and Joyce, 2023; Godet et al., 2019; Jing et al., 2019). Hypoxia-inducible factors, particularly hypoxia-inducible factor-1α (HIF-1α), act as central transcriptional regulators of cellular adaptation to oxygen deprivation. Under normoxic conditions, HIF-1α is hydroxylated by prolyl hydroxylase domain proteins (PHDs) and then recognized by the von Hippel-Lindau (VHL) E3 ubiquitin ligase complex, leading to proteasomal degradation. Under hypoxia, this hydroxylation process is inhibited, allowing HIF-1α to accumulate, translocate to the nucleus, heterodimerize with hypoxia-inducible factor-1β, recruit transcriptional coactivators such as p300/CREB-binding protein, and activate hypoxia-responsive genes (Nishigaki et al., 2025; Semenza, 2014; Yu et al., 2021; Lee et al., 2004). Beyond oxygen availability, HIF-1α activity can also be regulated through non-canonical mechanisms, including estrogen receptor β2-mediated stabilization, RING finger protein 20 - and VHL-associated ubiquitination, ubiquitin-specific protease 11 (USP11)-dependent deubiquitination, and ubiquitin-conjugating enzyme E2 S (UBE2S)-mediated VHL destabilization (Dey et al., 2015; Liu et al., 2025; Qiao et al., 2024; Lei and Lee, 2024). Together, these regulatory mechanisms underscore the complexity of HIF-1α activation in tumors, even under normoxic conditions.

Accumulating evidence indicates that HIF-1α promotes metastasis by activating invasion- and angiogenesis-related programs and by coordinating EMT, extracellular matrix (ECM) remodeling, vascular remodeling, stemness, and immune evasion. Elevated intratumoral HIF-1α expression is associated with tumor progression, metastatic potential, therapeutic resistance, and poor prognosis in multiple malignancies (Wicks and Semenza, 2022; Li Z. et al., 2024). Mechanistically, HIF-1α drives EMT-associated transcriptional programs and ECM remodeling, thereby facilitating local invasion (Wicks and Semenza, 2022; Rassouli et al., 2025). In hepatocellular carcinoma (HCC), hypoxia-induced HIF-1α cooperates with Ras homolog gene family member A/Rho-associated coiled-coil containing protein kinase and Ras-related C3 botulinum toxin substrate 1/p21-activated kinase signaling to promote vasculogenic mimicry and metastatic phenotypes (Zhang et al., 2020). Recent studies further suggest that hypoxia-associated signaling enhances metastasis through epigenetic and exosome-mediated mechanisms. For example, the HIF-1α/activating transcription factor 3 (ATF3)/P4HA1 axis promotes collagen deposition and breast cancer invasion, while hypoxia-induced SRY-box transcription factor 9 lactylation enhances glycolysis, stemness, migration, and invasion in non-small cell lung cancer (NSCLC) (Dhamdhere et al., 2025; Yan et al., 2024). In addition, CRC-derived exosomal microRNA-1825 and M2 macrophage-derived exosomal MALAT1 reinforce angiogenesis, glycolysis, metastasis, and chemoresistance (Sun et al., 2025a; Wang Y. et al., 2024).

Importantly, HIF-1α is now recognized as a central link between hypoxia, metabolic reprogramming, and tumor metastasis. Metabolic reprogramming is a hallmark of cancer progression, enabling tumor cells to adapt dynamically to the distinct nutrient, oxygen, redox, and energetic constraints encountered during local invasion, circulation, extravasation, and distant colonization (Bergers and Fendt, 2021; Faubert et al., 2020). Experimental platforms that recapitulate these features are also emerging; an oxygen-concentration-controllable multiorgan microfluidic system has been developed to model hypoxia-induced lung cancer-liver metastasis and to facilitate drug screening in a multicompartment setting (Zheng et al., 2021).

Hypoxia-associated metabolic remodeling, including increased glucose uptake and lactate production, may acidify the TME and contribute to immunosuppressive phenotypes. The extent of HIF-1α dependence varies by tumor context (Stegen et al., 2016; Park and Hall, 2025). Metabolic plasticity is particularly important because metastatic lesions often exhibit metabolic characteristics distinct from those of their corresponding primary tumors (Bergers and Fendt, 2021; Hindes et al., 2025). Moreover, metabolites are not merely passive products of cellular metabolism; lactate, adenosine, acetyl-CoA, and other metabolic intermediates can reshape epigenetic states and the immune microenvironment, facilitating immune escape and metastatic colonization. Metabolic reprogramming may also contribute to organ-specific metastasis, as exemplified by γ-aminobutyric acid accumulation-mediated remodeling of the astrocytic microenvironment in NSCLC brain metastasis (Xie et al., 2025).

Therefore, HIF-1α should be viewed not merely as a hypoxia-responsive transcription factor but as a central regulator of metabolic adaptation that integrates environmental stress signals with tumor cell plasticity and metastatic competence. In this review, we summarize the mechanisms by which HIF-1α regulates glucose, lipid, amino acid, nucleotide, and metabolite-associated metabolic pathways during tumor metastasis. We further discuss how HIF-1α-driven metabolic remodeling shapes the TME, promotes immune evasion and distant colonization, and provides potential metabolic vulnerabilities for the development of anti-metastatic therapeutic strategies.

### 1.1 Literature search strategy

This narrative review was based on searches of PubMed/MEDLINE, Web of Science Core Collection, supplemented by CNKI and Wanfang Data for Chinese-language literature. Records from database inception to September 2026 were covered, with priority given to studies published in the last 10 years; classic mechanistic studies were retained when relevant. Search terms combined MeSH and free-text words for hypoxia/HIF-1α, cancer/metastasis, and metabolic reprogramming, including glycolysis, lactylation, lipid metabolism, fatty acid oxidation, cholesterol metabolism, amino acid metabolism, glutamine metabolism, serine metabolism, TME, immune evasion, epithelial-mesenchymal transition, and therapeutic target. Corresponding Chinese terms were also used. Reference lists of included articles were checked for additional key studies. Inclusion criteria were peer-reviewed original articles, reviews, meta-analyses, and preclinical/clinical studies on the hypoxia/HIF-1α-cancer metabolism-metastasis axis. Duplicates, conference abstracts without full text, unrelated studies, and non-tumor studies lacking mechanistic relevance were excluded. Several hundred relevant publications were reviewed, and 142 articles were ultimately selected as references. As a narrative review, this work did not follow a systematic review protocol, and selection bias cannot be completely excluded.

### 1.2 Evidence grading

To facilitate comparison across preclinical and clinical studies, we graded evidence using the following hierarchy: E0, *in vitro* cell line or non-tumor model; E1, *in vivo* animal model without metastasis endpoint; E2, *in vivo* animal metastasis model; E3, patient-derived model; E4, observational human tumor study; E5, early-phase interventional clinical trial; E6, late-phase clinical trial or approved indication with proven anti-metastatic benefit; E6*, approval for another indication without proven anti-metastatic efficacy. When multiple evidence levels were available, the highest level was reported unless otherwise specified.

## 2 HIF-1α-mediated tumor metabolic reprogramming in metastasis: molecular mechanisms and regulatory roles

Intratumoral hypoxia is spatially and temporally heterogeneous, and chronic versus cyclic hypoxia may generate distinct metabolic, vascular, and immune phenotypes. This heterogeneity can limit drug penetration and the durability of small-molecule inhibition. Therefore, single-agent HIF-1α inhibition may be insufficient to achieve sustained anti-metastatic efficacy, given metabolic plasticity and intratumoral heterogeneity, and rational combination strategies are needed (Fallah and Rini, 2019). Emphasizing this heterogeneity before the pathway-specific sections avoids treating hypoxia as a homogeneous condition and provides a unified framework for the subsequent glucose, lipid, amino acid, and nucleotide metabolic pathways.

### 2.1 HIF-1α-regulated glucose metabolic reprogramming drives tumor metastasis

HIF-1α is a major driver of the Warburg effect in tumor cells. This metabolic shift not only supports the biosynthetic demands of rapid proliferation but also contributes to microenvironmental acidification that favors invasion and metastasis. Under hypoxia, HIF-1α transcriptionally activates glycolytic enzymes and glucose transporters, thereby reprogramming glucose metabolism to supply energy, biosynthetic precursors, and signaling metabolites required for metastasis. The following sections focus on glycolysis, lactate and pyruvate metabolism, and tricarboxylic acid (TCA) cycle intermediates.

#### 2.1.1 Glycolysis

Under physiological conditions, cellular adenosine triphosphate (ATP) is mainly generated through mitochondrial oxidative phosphorylation. However, even in the presence of oxygen, tumor cells preferentially convert glucose to lactate through aerobic glycolysis, known as the Warburg effect (Wicks and Semenza, 2022). Although less efficient in ATP production, this pathway supports the energy demands of rapid proliferation. Under hypoxia, HIF-1α also suppresses mitochondrial biogenesis and function, reducing reactive oxygen species (ROS) production and protecting tumor cells from oxidative damage (Zhang et al., 2007).

HIF-1α promotes adaptation to hypoxia by transcriptionally upregulating glycolytic genes. It increases the expression of pyruvate kinase M2 (PKM2), glucose transporter 1 (GLUT1), glucose transporter 3 (GLUT3), and lactate dehydrogenase A (LDHA), thereby enhancing glucose uptake and glycolytic flux (Wei et al., 2025; Zhao et al., 2014). Beyond these canonical targets, HIF-1α activates additional regulators that amplify glycolytic and metastatic programs. In breast cancer, HIF-1α directly binds the neuronal regeneration-related protein (NREP) promoter and induces NREP expression; NREP subsequently increases glucose consumption, ATP and lactate production, and the expression of GLUT1, hexokinase 2, PKM2, and LDHA, promoting proliferation, angiogenesis, EMT, migration, invasion, and lung metastasis (Ruan et al., 2024). In ovarian cancer, HIF-1α cooperates with phosphoinositide 3-kinase (PI3K)/protein kinase B (AKT)/mechanistic target of rapamycin (mTOR) signaling to promote glycolytic reprogramming, peritoneal metastasis, and immune evasion, supporting the relevance of HIF-1α-regulated glycolysis across multiple malignancies (Li et al., 2023). Notably, the glycolytic program is sensitive to hypoxia mode: cyclic acute hypoxia induces glycolytic gene expression more strongly than chronic hypoxia, including hexokinase 2 and phosphoglycerate kinase 1, whereas chronic hypoxia may favor sustained glycolytic flux and lactate export. This program mainly supports local invasion and circulatory survival.

Upstream regulators, bypass pathways, and feedback loops further modulate this pathway. TRIM65 promotes AMPK degradation and relieves AMPK-mediated suppression of HIF-1α (Li M. H. et al., 2025); sodium butyrate induces SIRT4 and autophagic degradation of HIF-1α (Zhang Q. et al., 2024); the miR-485-5p/NQO1 axis enhances glycolysis independently of direct HIF-1α regulation (Wang et al., 2025). In contrast, USP11 stabilizes HIF-1α and forms a bidirectional positive-feedback loop with PDK1/LDHA-dependent glycolysis (Qiao et al., 2024). These regulators can be classified as upstream regulators (TRIM65-AMPK, SIRT4), bypass pathways (miR-485-5p/NQO1), and bidirectional feedback loops (USP11-HIF-1α-PDK1/LDHA).

LDHA localization additionally influences lactate production. BAP1 interacts with LDHA and retains it in the nucleus, reducing cytoplasmic LDHA and lactate generation. BAP1 inactivation increases cytoplasmic LDHA accumulation, promotes pyruvate-to-lactate conversion, acidifies the TME, and facilitates immunosuppression and tumor progression (Zheng et al., 2024). HIF-1α also induces pyruvate dehydrogenase kinase 1 (PDK1), which phosphorylates and inactivates pyruvate dehydrogenase (PDH), blocking pyruvate conversion to acetyl-CoA and entry into the TCA cycle. This redirects metabolic flux from oxidative phosphorylation to glycolysis and promotes lactate production (Kim et al., 2006; Acuña-Pilarte and Koh, 2025). In HCC, USP11 stabilizes HIF-1α through deubiquitination, thereby promoting PDK1/LDHA-dependent glycolysis, proliferation, migration, and metastatic potential (Qiao et al., 2024).

HIF-1α-induced GLUT1 and glycolytic enzymes can also directly promote migration and invasion (Chen Z. et al., 2022). In esophageal cancer, GLUT1 binds epidermal growth factor receptor (EGFR) and prevents its ubiquitin-proteasome-mediated degradation, thereby suppressing autophagy-dependent ferroptosis, maintaining stem cell-like properties, and promoting tumor growth and lung metastasis (Sui et al., 2025). Similarly, GLUT1 binds phosphorylated EGFR in lung adenocarcinoma (LUAD), stabilizing EGFR signaling and promoting proliferation, migration, and invasion (Zhou et al., 2024). Glycolytic reprogramming also contributes to treatment-resistant phenotypes; GPRC5A modulates temozolomide resistance in glioblastoma through this mechanism (Alafate et al., 2025). From a therapeutic perspective, inhibition of core glycolytic nodes may trigger compensatory flux rewiring toward glutaminolysis or fatty acid oxidation to maintain energy supply; isotope tracing and dynamic flux analysis can be applied to characterize these compensatory pathways.

Aerobic glycolysis further generates intermediates for biosynthesis: 3-phosphoglycerate supports serine and glycine synthesis, whereas pyruvate can be transaminated to alanine. HIF-1α also affects the balance between glycolysis and the pentose phosphate pathway (PPP), thereby influencing redox homeostasis and survival under metastatic stress (Hayes et al., 2020). In a CoCl_2_-induced endothelial hypoxia model, HIF-1α stabilization reduced glucose-6-phosphate dehydrogenase expression and activity, whereas restoration of this enzyme reversed endothelial dysfunction, supporting a role for HIF-1α in PPP regulation (Yang et al., 2022). Lactate, the major glycolytic product, can serve as a carbon source for lipid biosynthesis rather than merely as a metabolic waste product (Du et al., 2022).

Collectively, HIF-1α-driven glycolysis promotes metastasis by increasing glucose uptake and glycolytic flux, generating lactate, supplying biosynthetic intermediates, and reinforcing EMT, stemness, dedifferentiation, and therapy resistance. Recent reviews further support the central role of this axis in metastatic progression (Zeng et al., 2025; Qiong et al., 2024).

In summary, the best-supported mechanism is HIF-1α-dependent upregulation of GLUT1, PKM2, GLUT3, and LDHA, which increases glucose uptake and glycolytic flux. Context-dependent findings include tumor-specific reliance on NREP, GLUT1/3, and PDK1, as well as upstream (TRIM65-AMPK, SIRT4) and bypass (miR-485-5p/NQO1) regulation. Evidence is strongest for core glycolytic enzymes, whereas several bypass pathways remain less validated. Key unresolved questions include how acute, cyclic, and chronic hypoxia differentially reshape glycolytic flux and compensatory resistance.

#### 2.1.2 Lactate

To survive the metastatic cascade, tumor cells must resist cell death, evade immune surveillance, and remodel the microenvironment. Lactate is a key effector metabolite that contributes to all three processes. Under HIF-1α regulation, tumor cells exhibit increased lactate production and export, together with enhanced lactate accumulation in the TME, uptake, and utilization, thereby establishing a lactate-dependent immunosuppressive phenotype. In hypoxic solid tumors, HIF-1α transcriptionally upregulates lactate dehydrogenase A, promoting lactate production (Yang et al., 2023). HIF-1α signaling also enhances monocarboxylate transporter 1/4 (MCT1/4)-dependent lactate transport and utilization, forming a reinforcing regulatory circuit between HIF-1α and lactate metabolism (Li S. et al., 2025). Notably, chronic hypoxia induces a sustained metabolic switch from oxidative phosphorylation to anaerobic glycolysis, leading to continuous lactate production and acidosis, whereas cyclic hypoxia enhances glycolysis through upregulation of glycolytic genes and monocarboxylate transporters that export lactate. Lactate accumulation in the TME can further stabilize HIF-1α, creating an oscillatory feedback that is modulated by extracellular lactate abundance. Lactate-mediated immunosuppression is most relevant to immune evasion and distant colonization.

These lactate-related mechanisms can be categorized into distinct regulatory modes. HIF-1α downstream effects include LDHA upregulation, BNIP3-dependent mitophagy, and SLC1A1-mediated ferroptosis resistance. MCT1/MCT4-dependent lactate transport and Cr (VI)-induced MCT1/MCT4 expression represent context-dependent or bypass mechanisms. Bidirectional feedback is exemplified by the lactate-ABCF1-K430la-HIF1A loop and by lactate accumulation that further increases HIF-1α expression. In contrast to glycolysis, canonical upstream regulators such as TRIM65-AMPK or SIRT4 have not been firmly established for lactate metabolism.

Mechanistically, lactate promotes metastasis through several pathways. First, lactate induces lysine 430 lactylation of ATP-binding cassette subfamily F member 1 (ABCF1). Lactylated ABCF1 retains its E2 ubiquitin-ligase activity, translocates to the nucleus, and activates lysine demethylase 3A (KDM3A) transcription, thereby stimulating the KDM3A-histone H3 lysine 9 dimethylation (H3K9me2)-HIF1A axis. This lactate-ABCF1-K430la-HIF1A positive-feedback loop amplifies HIF-1α signaling and promotes HCC growth and lung metastasis (Hong et al., 2025). In glioma, HIF-1α-regulated BNIP3-dependent mitophagy and histone lysine lactylation cooperate to mediate metabolic reprogramming, proliferation, and invasion (Dong et al., 2025). Second, lactate activates solute carrier family 1 member 1 (SLC1A1), increasing cystine uptake and glutathione synthesis, reducing lipid peroxidation, and conferring ferroptosis resistance in solid tumors (Yang et al., 2023). Third, in lung cancer cells, Cr(VI)-induced MCT1 and monocarboxylate transporter 4 (MCT4) expression enhances lactate utilization, while lactate accumulation further increases HIF-1α expression, reinforcing malignant phenotypes (Li S. et al., 2025). Therapeutically, lactate pathway inhibition may be compensated by upregulated glutamine catabolism or fatty acid oxidation, and combination strategies are required to overcome metabolic plasticity.

Beyond tumor cell-intrinsic effects, HIF-1α-driven lactate accumulation creates an acidic, immunosuppressive niche. Lactate impairs the proliferation and cytotoxic activity of CD8^+^ T cells and natural killer (NK) cells. Moreover, protein and histone lactylation, including histone H3 lysine 18 lactylation, promotes M2 macrophage polarization and myeloid-derived suppressor cell recruitment and activation, thereby facilitating immune evasion, invasion, and metastasis (Chen L. et al., 2022).

Clinically, high ABCF1-K430la expression in HCC correlates with poor prognosis and lung metastasis (Hong et al., 2025). Elevated LDHA expression and lactate accumulation in solid tumors are associated with ferroptosis resistance, immunosuppression, invasiveness, and metastatic risk (Yang et al., 2023). In lung cancer, co-high MCT1 and HIF-1α expression is associated with more rapid progression and increased metastasis (Li S. et al., 2025).

In summary, the best-supported mechanism is HIF-1α-driven LDHA upregulation and lactate production/export. Context-dependent findings include tumor-specific MCT1/MCT4 dependence and diverse cellular sources of lactate. Evidence is relatively strong for lactate-mediated immunosuppression and ABCF1-K430la feedback, but clinical causality remains incompletely defined. Key unanswered questions include how hypoxia dynamics affect lactate source, export, and uptake, and how lactate-targeted therapy should be combined with immune checkpoint blockade.

#### 2.1.3 Pyruvate

Pyruvate is a critical metabolic node linking glycolysis to the TCA cycle and influences tumor energy metabolism throughout metastasis. During invasion, extravasation, and distant colonization, tumor cells may reduce mitochondrial pyruvate oxidation and reconfigure oxidative phosphorylation, thereby adapting to hypoxic stress while maintaining aerobic glycolysis. Meanwhile, pyruvate and its downstream metabolites contribute to ECM remodeling and metastatic niche formation. Accordingly, pyruvate metabolic reprogramming is characterized by reduced mitochondrial pyruvate oxidation, increased conversion of pyruvate to lactate, and accumulation of pyruvate-derived or bypass metabolites that support invasion and colonization.

HIF-1α remodels pyruvate flux primarily by upregulating PDK1, thereby inhibiting PDH, while also influencing downstream pyruvate metabolism and propionate bypass pathways (Velpula et al., 2013). Notably, the pyruvate metabolic response is influenced by hypoxia mode: chronic hypoxia sustains PDK1-mediated PDH inhibition and a glycolytic shift, whereas cyclic hypoxia may modulate pyruvate flux through ROS-dependent mechanisms. Spatial hypoxic gradients within tumors can further create heterogeneous PDK1 activity and pyruvate utilization. This axis is primarily associated with local invasion, extravasation, and distant colonization.

Mechanistically, HIF-1α-induced PDK1 inhibits PDH, blocking mitochondrial pyruvate oxidation, maintaining aerobic glycolysis under hypoxia, and supporting tumor cell proliferation and invasion (Velpula et al., 2013). Environmental pyruvate taken up by tumor cells promotes α-ketoglutarate (α-KG) production and activates collagen prolyl-4-hydroxylase (P4HA), thereby increasing collagen hydroxylation, remodeling the ECM in distant organs such as the lungs, and facilitating metastatic niche formation, extravasation, and colonization (Elia et al., 2019). In addition, ERK2-mediated SP1/EGR1 regulation downregulates methylmalonyl-CoA epimerase, reducing propionate metabolic flux and causing methylmalonic acid accumulation in tumor cells and the TME; this metabolic alteration enhances invasiveness and metastatic potential (Gomes et al., 2022). Mechanistically, PDK1-PDH represents a direct HIF-1α downstream axis, whereas environmental pyruvate-P4HA and ERK2-SP1/EGR1-MCEE-methylmalonic acid pathways are indirect or bypass mechanisms. Targeting PDK1 alone may induce compensatory metabolic reprogramming, including enhanced glutaminolysis and fatty acid oxidation, which can be delineated by isotope tracing.

In glioblastoma, HIF-1α, PDK1, and EGFR are co-overexpressed, and combined PDK1/EGFR targeting reverses the glycolytic phenotype and suppresses tumor growth, supporting the tumor-promoting role of the HIF-1α/PDK1 axis (Velpula et al., 2013). In breast and lung cancer, low methylmalonyl-CoA epimerase expression and methylmalonic acid accumulation are associated with invasive and metastatic phenotypes (Gomes et al., 2022). In breast cancer, aberrant pyruvate metabolism promotes pulmonary metastatic niche formation and increases the risk of lung metastasis (Elia et al., 2019).

In summary, the best-supported mechanism is PDK1-mediated inhibition of PDH downstream of HIF-1α. Context-dependent findings include the indirect roles of environmental pyruvate-P4HA and ERK2-SP1/EGR1-MCEE-methylmalonic acid pathways. Evidence is strongest for PDK1-PDH, whereas propionate bypass regulation remains less clear. Key unresolved questions include whether propionate bypass is directly regulated by HIF-1α and how organ-specific pyruvate metabolism shapes metastasis.

#### 2.1.4 Tricarboxylic acid cycle

The TCA cycle is central to cellular energy metabolism, and abnormal accumulation of its intermediates can act as a signaling event that promotes tumor progression. HIF-1α directly or indirectly alters key TCA-cycle enzymes, disturbing metabolite homeostasis and creating conditions favorable for metastasis.

In CRC models, accumulation of succinate and fumarate directly inhibits PHD activity, stabilizes HIF-1α, and establishes a pseudohypoxic positive-feedback loop that amplifies pro-metastatic signaling (Ding et al., 2024). However, direct and indirect mechanisms should be distinguished: PHD inhibition and HIF-1α stabilization are supported by direct mechanistic evidence in the cited models, whereas altered TCA enzyme expression is often indirect or associative. These effects are context-dependent and are more prominent in chronic hypoxia and spatial hypoxic cores, whereas cyclic hypoxia may affect TCA enzymes through ROS. Tumor-derived succinate, fumarate, and α-KG may also be exchanged with immune cells and CAFs, shaping epigenetic and immune states. Organ-specific metastasis and therapy-resistance implications remain incompletely defined and require validation by isotope tracing. These effects are most relevant to colonization and dormant survival.

In summary, the best-supported mechanism is PHD inhibition and HIF-1α stabilization by succinate and fumarate. Context-dependent findings include tumor-type-specific TCA enzyme dependence and organ-specific associations such as CRC liver metastasis. Evidence is strongest for PHD inhibition/HIF-1α stabilization, whereas altered TCA enzyme expression is often indirect. Key unresolved questions include how chronic versus cyclic hypoxia affects TCA intermediate accumulation and whether compensatory FAO or glutaminolysis limits therapeutic efficacy.

#### 2.1.5 Succinate

During local invasion and distant colonization, tumor cells sustain hypoxia-responsive signaling and metabolite-driven malignant phenotypes through persistent succinate accumulation. This accumulation results from increased succinate production and impaired downstream catabolism. In chronically hypoxic regions, sustained HIF-1α activation may further amplify succinate accumulation through P4HA1-dependent signaling, whereas cyclic hypoxia may affect SDH activity through ROS-mediated mechanisms. Succinate accumulation is mainly associated with local invasion and liver colonization.

Under hypoxia, HIF-1α/ATF3-dependent P4HA1-succinate signaling has been implicated in glioblastoma progression (Yang et al., 2024). Separately, impairment of succinate dehydrogenase activity, including promoter hypermethylation-mediated silencing of SDHC in colorectal cancer, can lead to succinate accumulation. Accumulated succinate inhibits prolyl hydroxylase (PHD) activity, stabilizes HIF-1α, and induces EMT-associated transcription factors such as Snail and Slug, thereby promoting migration, invasion, and liver metastasis (Ding et al., 2024). Thus, enhanced succinate production and impaired degradation form a positive-feedback circuit that sustains tumor progression. SDH deficiency, SDHC promoter hypermethylation silencing, and low SDHB expression are upstream/bypass mechanisms; succinate inhibition of PHD stabilizing HIF-1α is a direct downstream/bidirectional feedback mechanism. From a therapeutic perspective, SDHC deficiency may support metastasis through compensatory fatty acid oxidation, which can be characterized by isotope tracing and dynamic flux analysis.

Clinical and experimental evidence indicates that P4HA1 overexpression in glioblastoma is accompanied by succinate accumulation, persistent HIF-1α activation, and increased malignancy (Yang et al., 2024). In CRC, reduced SDHB expression and succinate accumulation correlate with elevated HIF-1α levels and are more frequent in poorly differentiated, invasive, and metastatic tumors (Ding et al., 2024). Low SDHC expression is associated with worse prognosis and increased distant metastasis; SDHC deficiency may additionally support metastasis through fatty acid oxidation, indicating its dual role in CRC progression (Ding et al., 2024).

In summary, the best-supported mechanism is succinate accumulation leading to PHD inhibition and HIF-1α stabilization. Context-dependent findings include SDHB/SDHC evidence mainly from CRC and potential FAO-mediated compensation. Evidence is strongest for SDH deficiency/PHD inhibition, while clinical associations remain limited. Key unanswered questions include the downstream effectors of the HIF-1α/ATF3-P4HA1-succinate axis and organ specificity.

#### 2.1.6 Citrate

Citrate links glucose and lipid metabolism and supports local invasion and metastatic colonization. Tumor cells redirect citrate from mitochondrial oxidation toward cytosolic acetyl-CoA production, thereby supporting *de novo* lipogenesis, membrane biogenesis, energy homeostasis, and pro-metastatic signaling. This reprogramming is characterized by increased mitochondrial citrate export, expansion of the cytosolic citrate pool, enhanced citrate cleavage to acetyl-CoA, and attenuation of citrate-mediated negative regulation of HIF-1α.

HIF-1α-associated citrate reprogramming involves citrate transport, utilization, and metabolic diversion. Under hypoxia, HIF-1α promotes ATP-citrate lyase (ACLY) expression and cytosolic citrate cleavage, whereas hypoxic metabolic remodeling favors citrate export and utilization for lipid synthesis rather than mitochondrial oxidation (Zhang S. et al., 2025). In CRC, the mitochondrial citrate transporter SLC25A1 increases cytosolic citrate availability, supports *de novo* lipogenesis under basal conditions, and enhances oxidative phosphorylation under metabolic stress, thereby protecting tumor cells from energy stress-induced apoptosis (Yang et al., 2021). Notably, the effects of hypoxia on citrate metabolism may differ between chronic and cyclic hypoxia. Chronic hypoxia may favor sustained ACLY expression and citrate export for lipid synthesis, whereas cyclic hypoxia may modulate ACLY or SLC25A1 activity through ROS-dependent mechanisms, although this remains to be formally tested. Citrate reprogramming supports local invasion and peritoneal/lymph-node colonization.

Mechanistically, HIF-1α-mediated ACLY overexpression in gastric cancer promotes citrate cleavage to acetyl-CoA, diverting carbon from energy metabolism to *de novo* lipogenesis. This process supplies membrane components and signaling lipids and contributes to peritoneal metastasis (Zhang S. et al., 2025). SLC25A1 further supports CRC growth and survival by coordinating citrate export, lipid synthesis, and energy adaptation (Yang et al., 2021). Citrate also exerts context-dependent effects on HIF-1α signaling. In gastric cancer, exogenous citrate reduces HIF-1α and GLUT1 expression, indirectly suppresses phosphofructokinase activity, decreases lactate production and glycolytic flux, and induces apoptosis. However, ACLY overexpression may consume endogenous citrate and attenuate this inhibitory feedback. In low-glycolytic HCC, extracellular citrate induces ubiquitin-proteasome-dependent HIF-1α degradation, downregulates GLUT1 and LDHA, and inhibits hypoxic tumor growth (Kim et al., 2022). Exogenous citrate→HIF-1α degradation is upstream/bypass regulation, and ACLY consumption of citrate attenuating negative feedback is bidirectional feedback. Therapeutically, ACLY inhibition may trigger compensatory acetyl-CoA production from acetate via Acyl-CoA synthetase short-chain family member 2 (ACSS2), supporting metabolic plasticity and therapeutic resistance.

Clinically, ACLY is upregulated in gastric cancer tissues and peritoneal metastases, correlates positively with HIF-1α expression, and is associated with increased peritoneal metastasis and poor prognosis (Zhang S. et al., 2025). SLC25A1 expression is elevated in CRC relative to normal mucosa and correlates with tumor progression, lymph-node metastasis, and adverse prognosis (Yang et al., 2021). *In vitro* and *in vivo* studies further show that exogenous citrate inhibits gastric cancer and low-glycolytic HCC growth by suppressing HIF-1α signaling (Kim et al., 2022).

In summary, the best-supported mechanism is HIF-1α-ACLY/SLC25A1-driven citrate export and lipid synthesis. Context-dependent findings include opposing effects of exogenous citrate in different glycolytic backgrounds. Evidence is strongest for ACLY and SLC25A1 in mechanistic/preclinical models, whereas clinical associations are limited. Key unresolved questions include quantitative ACLY negative feedback and how citrate metabolism contributes to therapy resistance.

#### 2.1.7 α-Ketoglutarate (α-KG)

α-KG is a central TCA-cycle intermediate and an essential cofactor for PHDs and Jumonji C domain-containing histone demethylases (JMJDs). During local invasion and metastatic colonization, tumor cells must maintain HIF-1α stability, preserve hypoxic metabolic adaptation, and regulate epigenetic programs associated with EMT and angiogenesis. Accordingly, tumor cells may reduce α-KG availability, activate IDH2-dependent reductive carboxylation, and alter α-KG-dependent dioxygenase activity to favor metastatic gene expression. Under hypoxia, glutamine-derived α-KG is reductively carboxylated by IDH2 to form isocitrate, which can then be isomerized to citrate, supporting cell growth and viability. α-KG-dependent epigenetic reprogramming is linked to local invasion and distant colonization.

In CRC, IDH2-associated metabolic reprogramming has been reported to alter α-KG availability and may thereby affect PHD-dependent HIF-1α stability (Liu S. et al., 2024). Whether HIF-1α directly induces IDH2 expression requires further mechanistic validation. Reduced α-KG limits PHD activity, decreases HIF-1α hydroxylation and degradation, and consequently reinforces HIF-1α signaling. Altered α-KG availability may also affect the activity of α-KG-dependent PHDs and JMJDs, linking metabolic reprogramming to epigenetic regulation (Liu S. et al., 2024; Xiong et al., 2023). Low α-KG→PHD inhibition→HIF-1α stabilization is direct downstream/bidirectional feedback; low α-KG→JMJD inhibition→relief of Snail repression is indirect/epigenetic bypass; whether IDH2 is directly induced by HIF-1α remains to be validated. IDH2 inhibition may induce compensatory upregulation of other TCA cycle enzymes, requiring combination strategies to achieve sustained efficacy.

In CRC, IDH2 overexpression has been associated with altered α-KG metabolism and enhanced HIF-1α signaling (Liu S. et al., 2024). Insufficient α-KG impairs PHD-mediated HIF-1α degradation, allowing HIF-1α stabilization, glycolytic gene activation, and enhanced proliferation and invasion. Conversely, genetic or pharmacological IDH2 inhibition increases α-KG, promotes HIF-1α hydroxylation and degradation, impairs mitochondrial ATP production, and suppresses glycolysis and tumor growth (Liu S. et al., 2024). During breast cancer metastasis, low α-KG inhibits both PHD and JMJD activity. Reduced PHD activity stabilizes HIF-1α and activates EMT- and angiogenesis-related genes, whereas reduced JMJD activity relieves repression of metastasis-associated genes, including Snail, thereby enhancing migration and invasion (Xiong et al., 2023).

Clinical and experimental data show that IDH2 expression is increased in CRC and correlates with tumor progression, lymph-node metastasis, and poor prognosis (Liu S. et al., 2024). In breast cancer, α-KG metabolic imbalance and abnormal α-KG-dependent enzyme activity are associated with metastatic potential (Xiong et al., 2023). Inhibition of IDH2 or supplementation with exogenous α-KG suppresses tumor-cell proliferation, migration, and invasion through attenuation of HIF-1α signaling, indicating potential therapeutic value (Liu S. et al., 2024; Xiong et al., 2023).

In summary, the best-supported mechanism is low α-KG-mediated PHD inhibition and HIF-1α stabilization. Context-dependent findings include the unproven direct regulation of IDH2 by HIF-1α and dual effects on PHD and JMJD. Evidence is strongest for IDH2/PHD/JMJD in preclinical models, while clinical associations remain limited. Key unresolved questions include separating α-KG-dependent epigenetic regulation from HIF-1α stability and defining organ-specific roles.

In summary, HIF-1α reshapes glucose metabolism by promoting glycolysis through GLUT1, PKM2, and LDHA and by coordinating the flux of TCA-cycle intermediates, including succinate, citrate, and α-KG. The associated regulatory mechanisms and pro-metastatic nodes are summarized in Figure 1.

**FIGURE 1:**
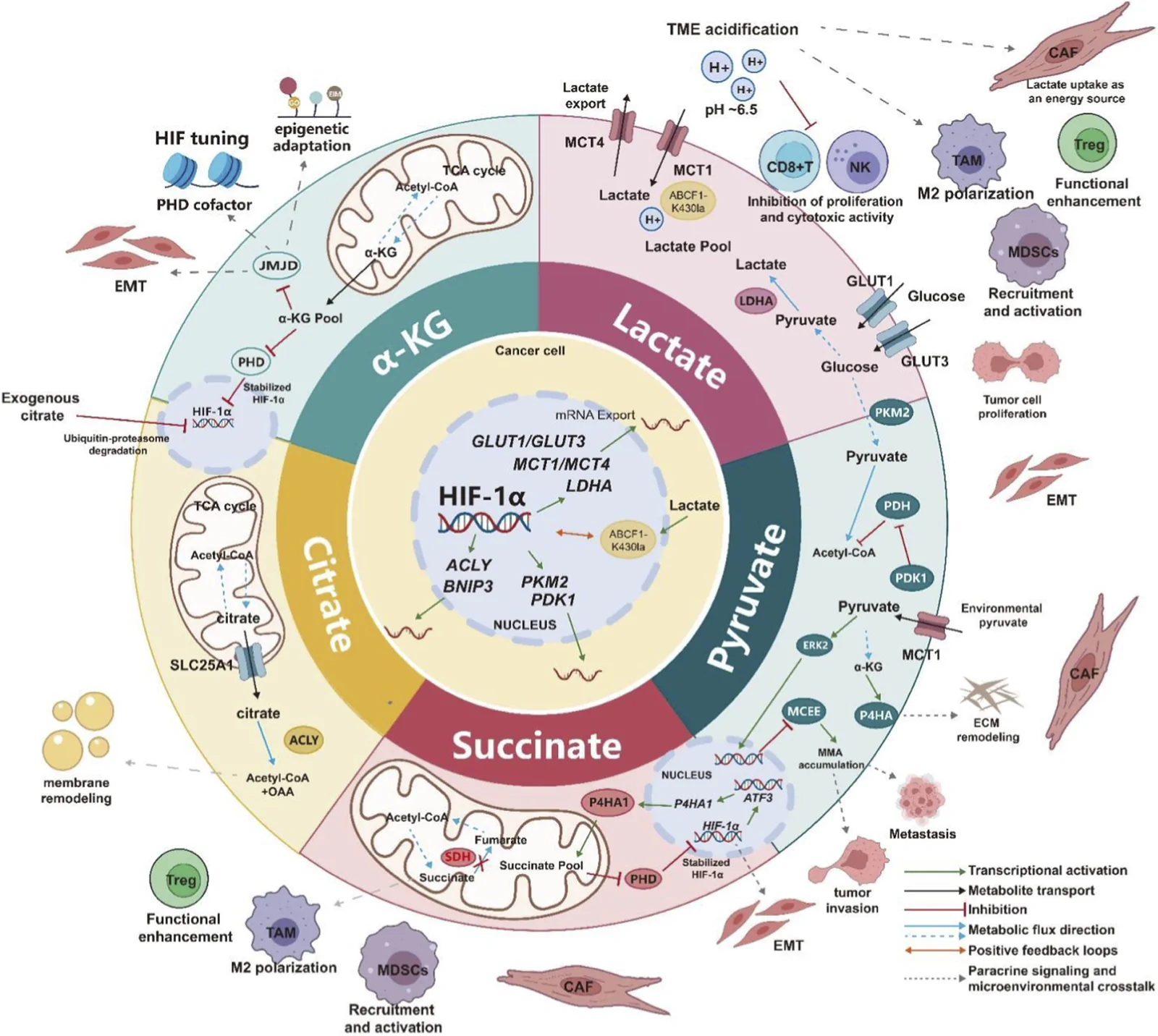
HIF-1α-mediated glucose and TCA-cycle metabolic reprogramming in hypoxia-driven tumor metastasis. Hypoxia-stabilized HIF-1α transcriptionally activates GLUT1/GLUT3, PKM2, LDHA, PDK1, MCT1/MCT4, ACLY and TCA-cycle regulators, driving glycolysis, lactate/pyruvate exchange, citrate/acetyl-CoA metabolism, and succinate/fumarate/α-KG accumulation. Arrows: green solid, HIF-1α transcriptional activation; black solid, metabolite transport; red T-bar, inhibition; blue dashed, metabolic flux; orange dashed, positive feedback; grey dotted, paracrine signaling and tumor-microenvironment crosstalk. Metabolic outputs link to EMT, immune evasion, ECM remodeling, pre-metastatic niche formation and metastasis. Crosstalk modules depict cancer cells, CAFs, TAMs, MDSCs, CD8^+^ T cells, NK cells and Tregs.

### 2.2 HIF-1α-regulated lipid metabolic reprogramming drives tumor metastasis

Lipid metabolic reprogramming is another major component of HIF-1α-driven tumor metastasis. In contrast to glucose metabolism, which mainly provides rapid energy and intermediates, lipid metabolism supplies membrane phospholipids, supports energy storage in lipid droplets, and generates signaling molecules such as fatty acid derivatives and acetyl-CoA. Under hypoxia, HIF-1α promotes tumor-cell invasion, survival, and distant colonization by coordinating fatty acid synthesis and oxidation, cholesterol metabolism, phospholipid remodeling, and ketone-body utilization. It should be noted that lipid metabolic responses are also context-dependent; chronic hypoxia favors lipid storage and suppression of fatty acid oxidation, whereas cyclic hypoxia may promote ROS-dependent lipid remodeling. As in glucose metabolism, HIF-1α-driven lipid metabolic reprogramming can be classified into direct downstream targets, upstream or bypass pathways, and bidirectional feedback loops, as detailed in each subsection.

HIF-1α regulates the expression of lipid metabolism-related genes, thereby modulating lipid synthesis, degradation, and storage to facilitate hypoxic adaptation, tumor growth, and survival. During malignant progression, lipid metabolic reprogramming promotes intracellular lipid accumulation, providing energy and macromolecular substrates for membrane synthesis and signal transduction (Li X. et al., 2024). Lipid accumulation also promotes lipid-droplet formation, which protects tumor cells from oxidative stress. Crosstalk between lipid droplets and intracellular free fatty acids (FFAs) can further establish an inflammatory microenvironment favorable for tumor growth (Cui et al., 2024).

HIF-1α affects lipid metabolism by regulating fatty acid synthesis, β-oxidation, and lipid-droplet formation. In particular, HIF-1α upregulates fatty acid synthase (FASN), thereby promoting *de novo* fatty acid synthesis (Lee et al., 2015; Guo et al., 2021). These changes increase the availability of lipids for membrane biogenesis and signaling. In addition, lipid metabolites, including prostaglandins and leukotrienes, may regulate interactions between tumor cells and stromal cells (Wang and Dubois, 2010). FASN may also directly promote tumor-cell migration by regulating cytoskeletal reorganization and membrane fluidity (Xu et al., 2021).

#### 2.2.1 Fatty acids

During the metastatic cascade, tumor cells require fatty acids for energy production, membrane biosynthesis, redox homeostasis, and signaling at the stages of invasion, migration, angiogenesis, and distant colonization. Accordingly, tumor cells often favor fatty acid storage and synthesis, although this pattern varies among tumor types and microenvironments. Fatty acid metabolic reprogramming primarily supports local invasion, angiogenesis, and distant colonization.

HIF-1α plays a central but context-dependent role in fatty acid metabolic reprogramming. At the intercellular communication level, extracellular vesicles released by mature adipocytes induce HIF-1α activity *in vitro* and *in vivo*, enhancing growth, motility, invasion, stemness, and EMT in estrogen receptor-positive and triple-negative breast cancer cells (La Camera et al., 2021). This adipocyte-derived EV-mediated regulation represents an upstream or microenvironmental bypass mechanism, rather than direct HIF-1α downstream signaling. In ccRCC, VHL loss stabilizes HIF and represses carnitine palmitoyltransferase 1A (CPT1A), reducing fatty acid transport into mitochondria and redirecting fatty acids toward lipid-droplet storage. These lipid droplets provide energy and biosynthetic precursors during nutrient deprivation, whereas increased CPT1A expression limits tumor growth (Du et al., 2017).

In contrast, in CRC, transforming growth factor (TGF)-β1 upregulates Acyl-CoA synthetase long-chain family member 3 (ACSL3) through SREBP1 signaling, activating fatty acid oxidation (FAO) and generating ATP and nicotinamide adenine dinucleotide phosphate (NADPH). NADPH maintains redox homeostasis and supports EMT and distant metastasis (Quan et al., 2022). In breast cancer, CPT1A-mediated FAO promotes proliferation, migration, and angiogenesis, while HIF-1α directly enhances CPT1A transcription. This CPT1A/HIF-1α positive-feedback loop reduces L-ascorbic acid uptake and increases extracellular L-ascorbic acid, which promotes angiogenesis through Nrf2/NQO1-mediated redox remodeling (Ma et al., 2025). Mechanistically, CPT1A/HIF-1α constitutes a bidirectional feedback loop rather than a simple downstream axis: HIF-1α transcriptionally upregulates CPT1A, and CPT1A in turn regulates HIF-1α ubiquitination.

Notably, fatty acid metabolic responses differ by hypoxia mode. Chronic hypoxia favors sustained suppression of FAO and lipid-droplet accumulation, whereas cyclic hypoxia may promote ROS-dependent lipid remodeling and metabolic plasticity, although the precise mechanisms remain incompletely defined.

Under hypoxia, HIF-1α upregulates Perilipin 2, FASN, and SREBP1 while downregulating CPT1 in CRC cells. This coordinated response enhances lipid uptake and *de novo* synthesis, suppresses fatty acid oxidation, and elevates intracellular FFA levels. Excess FFAs activate canonical Wnt signaling, promote β-catenin nuclear translocation, and accelerate CRC proliferation and progression (Ni et al., 2017). HIF-1α has been reported to promote FASN expression through a hypoxia-response element, independently of SREBP1 and simultaneously induce GLUT3 expression. The IGF1/Insulin-IGF1R-AKT-mTOR axis promotes HIF-1α accumulation, whereas FASN enhances NRF2-dependent antioxidant capacity and GLUT3 promotes hypoxic tolerance, together conferring dual stress adaptation in CRC cells (Wang et al., 2026).

In lung cancer, HIF-1α upregulates stearoyl-CoA desaturase 1 (SCD1). By altering membrane lipid composition, SCD1 activates PI3K/AKT/mTOR signaling and promotes proliferation. SCD1-derived fatty acids also induce lipid-droplet accumulation in cancer-associated fibroblasts (CAFs). Following lipid mobilization, CAF-derived fatty acids enter tumor-cell mitochondria for β-oxidation, providing energy for invasion and metastasis and further promoting EMT and Wnt/β-catenin signaling (Zhang et al., 2022). Thus, HIF-1α-mediated FASN activation and SCD1-dependent tumor-stroma lipid crosstalk support invasion and metastatic outgrowth (Ni et al., 2017; Zhang et al., 2022). From a therapeutic perspective, inhibition of FASN or SCD1 may trigger compensatory upregulation of alternative lipid synthesis pathways or a shift toward FAO-dependent energy supply; isotope tracing and dynamic flux analysis can be applied to characterize these compensatory pathways.

In summary, the best-supported mechanism is HIF-1α-dependent upregulation of FASN and SCD1, which promotes *de novo* fatty acid synthesis and lipid-droplet accumulation. Context-dependent findings include tumor-specific reliance on CPT1A-mediated FAO versus lipid storage, and the involvement of adipocyte-derived Extracellular vesicles (EVs) as upstream/bypass regulation. Evidence is strongest for FASN and SCD1 as direct HIF-1α targets, whereas the CPT1A/HIF-1α bidirectional feedback loop is supported by mechanistic studies in breast cancer. Key unresolved questions include how chronic versus cyclic hypoxia differentially regulates fatty acid synthesis and oxidation, and whether compensatory lipid metabolic rewiring limits the efficacy of FASN or SCD1 inhibition.

#### 2.2.2 Acetyl-CoA

Acetyl-CoA is a central metabolic hub linking glucose and lipid metabolism and contributes to local invasion, distant colonization, metastatic outgrowth, and immune evasion. Tumor cells require acetyl-CoA to support membrane biosynthesis, epigenetic activation of EMT- and angiogenesis-related genes, metabolic crosstalk with the microenvironment, and survival under metabolic stress. Accordingly, metastatic tumor cells may enhance ACLY-dependent acetyl-CoA synthesis and acetate uptake, reduce acetyl-CoA clearance, selectively suppress FAO-derived acetyl-CoA production, and expand the nuclear-cytosolic acetyl-CoA pool to meet biosynthetic and epigenetic demands. Acetyl-CoA metabolic reprogramming primarily supports local invasion, epigenetic reprogramming during EMT, immune evasion, and metastatic outgrowth.

HIF-1α regulates acetyl-CoA production and utilization at multiple levels. It directly upregulates ACLY, promoting endogenous acetyl-CoA synthesis (Ma et al., 2023), while HIF-1α-mediated suppression of medium-chain acyl-CoA dehydrogenase (MCAD) and long-chain acyl-CoA dehydrogenase (LCAD) through PGC-1β reduces acetyl-CoA production from fatty acid oxidation (Huang et al., 2014). Mechanistically, ACLY is a direct HIF-1α downstream transcriptional target, whereas MCAD/LCAD suppression and ACOT12 repression represent indirect or downstream-adjacent mechanisms. In HCC, tumor-associated macrophage (TAM)-derived acetate has been reported to serve as a metabolic reservoir that supports acetyl-CoA availability and metastatic behavior (Shen et al., 2025). In addition, indirect repression of acyl-CoA thioesterase 12 (ACOT12) impairs acetyl-CoA degradation and maintains elevated intracellular acetyl-CoA levels (Lu et al., 2019).

Notably, acetyl-CoA production from different sources may respond differently to hypoxia mode. Chronic hypoxia favors ACLY-dependent acetyl-CoA synthesis and sustained histone acetylation, whereas cyclic hypoxia may promote acetate uptake from the microenvironment as a compensatory route.

In ccRCC, HIF-1α-mediated ACLY upregulation promotes cytosolic citrate cleavage to acetyl-CoA, providing substrates for fatty acid synthesis and supporting EMT and lung metastatic colonization. HIF-1α knockdown or ACLY inhibition significantly reduces invasion and metastatic lesion formation *in vivo* (Ma et al., 2023). Similarly, in gastric cancer, HIF-1α-induced ACLY promotes acetyl-CoA, fatty acid, and membrane-lipid synthesis, thereby supporting proliferation, angiogenesis, EMT, invasion, and peritoneal metastasis (Zhang S. et al., 2025).

In HCC, hypoxia-induced HIF-1α upregulates USP13. USP13 deubiquitinates ACLY at K726, prevents its ubiquitin-proteasome-mediated degradation, and stabilizes ACLY protein. Stabilized ACLY promotes acetyl-CoA production and cholesterol metabolic reprogramming, enhances ferroptosis resistance, and reduces CD8^+^ T-cell infiltration, thereby facilitating immune evasion (Hu et al., 2025). In liver cancer, HIF-1α-induced lactate secretion activates lipid peroxidation in TAMs. ALDH2 subsequently converts lipid peroxides into acetate, which is released and taken up by tumor cells. The resulting increase in acetyl-CoA and histone acetylation activates EMT-related genes and promotes migration and distant metastasis (Shen et al., 2025).

Although HIF-1α-mediated suppression of MCAD and LCAD reduces FAO-derived acetyl-CoA, this effect can be compensated by ACLY-dependent acetyl-CoA synthesis. MCAD/LCAD co-suppression also reduces FAO-derived ROS, thereby supporting survival and proliferation under hypoxia. In addition, LCAD repression alone reduces PTEN expression and enhances metastatic potential (Huang et al., 2014). In HCC, low ACOT12 expression impairs acetyl-CoA degradation, resulting in acetyl-CoA accumulation. As a histone-acetylation donor, acetyl-CoA promotes H3K27ac enrichment at the Snail and Twist promoters, induces EMT, and enhances migration and invasion (Lu et al., 2019).

Acetyl-CoA-dependent histone acetylation may link metabolic state to PD-L1 expression and immune evasion in specific tumor models; the contribution of HIF-1α/ACLY signaling requires model-specific validation (Shurin and Umansky, 2022; Wang H. et al., 2024). Increased nuclear-cytosolic acetyl-CoA provides substrate for p300-mediated H3K27ac enrichment at the programmed death-ligand 1 (PD-L1) promoter, increasing PD-L1 transcription. PD-L1 subsequently interacts with programmed cell death protein 1 (PD-1) on T cells and suppresses T-cell activation and cytotoxicity. More broadly, reciprocal crosstalk between HIF signaling and the PD-1/PD-L1 axis can reinforce hypoxia-associated immune evasion and influence therapeutic responses to immune checkpoint blockade (Shurin and Umansky, 2022). Together with the TAM-derived acetate-acetyl-CoA pathway, this mechanism couples epigenetic reprogramming, immune evasion, and metastatic progression (Shen et al., 2025; Wang H. et al., 2024). Therapeutically, ACLY inhibition may trigger compensatory acetyl-CoA production from acetate via ACSS2 or from FAO-derived sources, supporting metabolic plasticity and therapeutic resistance.

Clinical and experimental evidence shows that ACLY overexpression in ccRCC is associated with EMT, lung metastasis, and poor prognosis (Ma et al., 2023). In gastric cancer, ACLY is upregulated in primary tumors and peritoneal metastases, and high ACLY expression correlates with increased peritoneal dissemination and shorter survival (Zhang S. et al., 2025). In HCC, USP13 overexpression is associated with poor prognosis, whereas the USP13 inhibitor 2-methoxyestradiol induces ferroptosis and enhances T cell-mediated cytotoxicity in patient-derived organoids and animal models (Hu et al., 2025). Elevated tumor lactate levels correlate with TAM infiltration and distant metastasis in liver cancer (Shen et al., 2025). Low MCAD and LCAD expression is associated with enhanced proliferation and increased lung-metastasis risk (Huang et al., 2014). In melanoma, ACLY overexpression correlates with elevated PD-L1 expression, poor immune-checkpoint inhibitor efficacy, and unfavorable prognosis (Wang H. et al., 2024). Low ACOT12 expression is also associated with high metastatic risk and poor prognosis in liver cancer, whereas ACOT12 overexpression suppresses metastatic ability *in vivo* (Lu et al., 2019).

In summary, the best-supported mechanism is HIF-1α-dependent ACLY upregulation and acetyl-CoA production, which supports membrane biosynthesis, histone acetylation, and immune evasion. Context-dependent findings include the involvement of TAM-derived acetate, MCAD/LCAD suppression, and ACOT12 repression as indirect or microenvironmental mechanisms. Evidence is strongest for ACLY as a direct HIF-1α target, whereas the contribution of the TAM-acetate pathway is supported mainly by HCC studies. Key unresolved questions include how hypoxia mode affects the relative contribution of different acetyl-CoA sources, and whether compensatory acetate uptake or FAO-derived acetyl-CoA limits the efficacy of ACLY inhibition.

#### 2.2.3 Cholesterol

During local invasion, distant colonization, and metastatic outgrowth, tumor cells require increased cholesterol for membrane biogenesis, lipid-raft assembly, and survival in quiescent states. They therefore exhibit enhanced cholesterol synthesis, uptake, esterification, stromal lipid transfer, and cholesterol-HIF-1α feedback signaling. Cholesterol metabolic reprogramming primarily supports local invasion, dormant survival, and lung colonization.

HIF-1α regulates cholesterol metabolism through multiple mechanisms. In melanoma, high scavenger receptor SR-B1 expression is associated with increased cutaneous invasiveness. SR-B1 deletion reduces HIF1A expression, In melanoma, SR-B1, cholesterol metabolism, and HIF-1α signaling have been reported to be interconnected, although the directionality and directness of these relationships remain context dependent (Eckel et al., 2025). This SR-B1-HIF-1α relationship represents a context-dependent association rather than a firmly established bidirectional feedback loop. In lung cancer models, SCD1-dependent lipid-droplet accumulation in CAFs has been implicated in tumor-stroma metabolic crosstalk and disease progression (Zhang et al., 2022).

Notably, hypoxia-mediated regulation of cholesterol synthesis is graded relative to oxygen availability and can occur independently of HIF. SREBP2 degradation and 3-hydroxy-3-methylglutaryl-CoA reductase (HMGCR) suppression in severe hypoxia are mediated by Membrane ring finger 6 and occur even when HIF signaling is abolished, indicating that HIF-independent pathways also contribute to cholesterol metabolic remodeling under hypoxia.

During metastatic colonization, quiescent HCC cells induced by high-density culture upregulate Sterol regulatory element-binding protein-2 (SREBP2) and the cholesterol efflux transporter ATP-binding cassette subfamily G member 1. Cholesterol supports the survival of quiescent cells and contributes to lung colonization following reactivation (Liu X. et al., 2024). In CRC, SREBP1 overexpression increases ROS levels, NF-κB-p65 phosphorylation, and MMP7 expression, thereby promoting angiogenesis, invasion, and metastasis (Gao et al., 2019). FABP4 overexpression may further promote CRC migration and invasion by enhancing fatty acid transport, activating AKT signaling, and inducing EMT (Tian et al., 2020).

In HCC, RPL6 stabilizes 3-hydroxy-3-methylglutaryl-CoA synthase 1 (HMGCS1) mRNA and increases intracellular cholesterol. Cholesterol then binds PHD2, inhibits its activity, reduces HIF-1α hydroxylation and VHL-mediated degradation, and stabilizes HIF-1α. The resulting RPL6-HMGCS1-cholesterol-HIF-1α positive-feedback axis activates angiogenesis-, invasion-, migration-, and metabolic reprogramming-related genes, thereby promoting HCC metastasis (Yang et al., 2025). This RPL6-HMGCS1-cholesterol-HIF-1α axis constitutes a bidirectional feedback loop: cholesterol stabilizes HIF-1α, and HIF-1α-driven metabolic reprogramming may further reinforce cholesterol synthesis. In addition, sterol O-acyltransferase 1 (SOAT1) promotes cholesterol esterification, increases plasma-membrane cholesterol and cholesteryl-ester accumulation, activates Transforming growth factor-β(TGF-β)/MAPK, Wnt/β-catenin, and PI3K/AKT/mTOR signaling, and induces EMT in HCC (Fu et al., 2024). Therapeutically, inhibition of cholesterol synthesis or SOAT1 may trigger compensatory cholesterol uptake via SR-B1 or LDLR, supporting metabolic plasticity and therapeutic resistance.

Clinical evidence indicates that SR-B1 overexpression in melanoma is associated with increased cutaneous invasiveness (Eckel et al., 2025), whereas the HIF-1α/SCD1 axis in lung-cancer CAFs represents a potential therapeutic target (Zhang et al., 2022). Cholesterol metabolic reprogramming in quiescent HCC cells is associated with increased lung-metastatic propensity (Liu X. et al., 2024), and SREBP1 overexpression in CRC correlates with invasion and metastasis (Gao et al., 2019). Activation of the RPL6-HMGCS1-cholesterol-HIF-1α axis and SOAT1 overexpression in HCC are associated with high metastatic risk and poor prognosis (Yang et al., 2025; Fu et al., 2024).

In summary, the best-supported mechanism is the RPL6-HMGCS1-cholesterol-HIF-1α bidirectional feedback loop, which stabilizes HIF-1α and promotes HCC metastasis. Context-dependent findings include the HIF-independent, Membrane ring finger 6-mediated degradation of SREBP2 and HMGCR in severe hypoxia, as well as SR-B1-HIF-1α associations that remain directionally unresolved. Evidence is strongest for the cholesterol-PHD2-HIF-1α feedback axis in HCC, whereas the role of SREBP2 in hypoxic cholesterol regulation appears to be largely HIF-independent. Key unresolved questions include how chronic versus cyclic hypoxia differentially affects cholesterol synthesis and uptake, and whether compensatory cholesterol uptake limits the efficacy of cholesterol synthesis inhibition.

#### 2.2.4 Phospholipids

During local invasion, distant colonization, and metastatic outgrowth, tumor cells must rapidly expand their membranes and migrate through confined tissue spaces. They therefore enhance phospholipid synthesis, remodel membrane phospholipid composition, and increase polyunsaturated fatty acid (PUFA) incorporation to maintain membrane biogenesis, fluidity, and plasticity. Phospholipid metabolic reprogramming primarily supports local invasion, confined migration, and EMT-associated plasticity.

In lipid-rich ovarian cancer microenvironments, adipocyte-derived signals can redirect glucose-derived carbon toward G3P synthesis, thereby increasing glycerophospholipid and triacylglycerol synthesis (Mukherjee et al., 2023). This adipocyte-driven redirection of glucose carbon represents a microenvironmental bypass mechanism rather than direct HIF-1α transcriptional regulation. In gastric cancer, hypoxic HIF-1α directly upregulates CEPT1, a key enzyme in phosphatidylcholine (PC) synthesis. Increased PC accumulation enhances membrane fluidity, plasticity, anti-apoptotic capacity, and confined migration (Zhou et al., 2025).

Notably, the phospholipid response to hypoxia may differ by hypoxia mode: chronic hypoxia favors sustained CEPT1/PC-mediated membrane remodeling, whereas cyclic hypoxia may promote intermittent phospholipid desaturation and PUFA incorporation through ROS-dependent mechanisms, although this requires further validation.

During EMT, lipid desaturation can shift from SCD dependence toward fatty acid desaturase 2 dependence. Fatty acid desaturase 2 and β-catenin form a positive feedback loop that increases the PUFA proportion in membrane phospholipids, thereby enhancing membrane fluidity and mesenchymal-like migration. This phospholipid remodeling is important for maintaining EMT plasticity and promoting invasion and metastasis. However, although SCD inhibition can induce apoptosis, it may also activate AMPK and increase TGF-β2 expression, potentially enhancing survival plasticity (Chen et al., 2025). Mechanistically, FADS2/β-catenin constitutes a positive-feedback loop that promotes phospholipid desaturation and EMT plasticity.

Studies show that HIF-1α-driven G3P synthesis is associated with enhanced metastatic capacity in lipid-rich ovarian cancer microenvironments (Mukherjee et al., 2023). In gastric cancer, HIF-1α/CEPT1 activation correlates with increased PC content and enhanced confined migration (Zhou et al., 2025). Fatty acid desaturase 2-dependent phospholipid desaturation is an important EMT-associated metabolic adaptation; therefore, compensatory survival responses should be considered when applying SCD inhibitors clinically (Chen et al., 2025). Therapeutically, SCD inhibition may trigger compensatory FADS2-dependent desaturation and AMPK-mediated survival signaling, supporting metabolic plasticity and limiting the efficacy of single-agent SCD inhibition.

In summary, the best-supported mechanism is HIF-1α-dependent CEPT1 upregulation and PC synthesis, which enhances membrane fluidity and confined migration in gastric cancer. Context-dependent findings include the shift from SCD to FADS2-dependent desaturation during EMT and the involvement of adipocyte-derived G3P as a bypass mechanism. Evidence is strongest for the HIF-1α/CEPT1/PC axis, whereas the FADS2/β-catenin feedback loop is supported mainly by EMT model studies. Key unresolved questions include how chronic versus cyclic hypoxia affects phospholipid desaturation and whether compensatory FADS2 activation limits the efficacy of SCD inhibitors.

#### 2.2.5 Ketone bodies

During invasion and distant colonization, tumor cells require angiogenesis to maintain oxygen and nutrient supply while adapting to glucose deprivation in metastatic lesions. Ketone bodies have context-dependent effects in this process. Exogenous β-hydroxybutyrate (BHB) can suppress HIF-1α signaling and angiogenesis, whereas tumor cells may enhance ketone-body utilization to acquire alternative energy sources and biosynthetic precursors through metabolic cooperation with stromal cells. Ketone-body metabolic reprogramming primarily supports local invasion, angiogenesis, and lung colonization.

BHB directly targets HIF-1α, reduces VEGFA transcriptional activity, and exerts anti-angiogenic effects (Huang et al., 2024). This BHB-mediated suppression of HIF-1α represents an upstream or counter-regulatory mechanism, rather than a HIF-1α downstream effect. However, HCC cells can reactivate ketone-body catabolism. Under IGF1 stimulation, ERK2 phosphorylates SUCLA2 at S124, after which PIN1-mediated cis-trans isomerization facilitates SUCLA2 binding to OXCT1. SUCLA2-derived succinyl-CoA provides substrate for OXCT1 and succinylates OXCT1 at K421, increasing its enzymatic activity. This process promotes ketolysis and acetyl-CoA production for the TCA cycle, supporting HCC proliferation and tumor growth (Guo et al., 2025). This SUCLA2-OXCT1 axis represents a metabolic bypass mechanism that enhances ketolysis independently of direct HIF-1α transcriptional regulation.

Notably, ketone-body utilization may be differentially regulated by hypoxia mode. In glioma cells, hypoxia downregulates OXCT1, Acetyl-CoA acetyltransferase 1, and β-hydroxybutyrate dehydrogenase 1/2 expression, suggesting that chronic hypoxia may suppress ketone-body catabolism in certain tumor types, whereas in HCC, IGF1-driven OXCT1 activation can support ketolysis under metabolic stress.

The two-compartment tumor-metabolism model further illustrates metabolic cooperation between CAFs and tumor cells. CAFs expressing HMGCS2 and β-hydroxybutyrate dehydrogenase 1 produce and release ketone bodies, including BHB. Tumor cells upregulate ketone-body utilization enzymes, such as OXCT1/2 and Acetyl-CoA acetyltransferase 1/2, to convert imported ketone bodies into acetyl-CoA for mitochondrial biogenesis and lipid synthesis. In MDA-MB-231 breast cancer cells, overexpression of ketone-body utilization enzymes enhances invasiveness and lung metastatic colonization (Martinez-Outschoorn et al., 2012). This CAF-tumor ketone-body coupling represents a paracrine metabolic cooperation mechanism, rather than a cell-intrinsic HIF-1α downstream program.

Clinical and experimental evidence indicates that reduced BHB levels in the colonic mucosa of patients with inflammatory bowel disease are associated with increased CRC-progression risk. In the AOM/DSS colitis-associated CRC model, exogenous BHB reduces tumor burden and lung-metastasis formation (Huang et al., 2024). Increased ketone-body utilization is also associated with aggressive phenotypes and metastatic potential in breast cancer models (Martinez-Outschoorn et al., 2012), and CAF-tumor-cell ketone-body metabolic coupling enhances lung colonization (Martinez-Outschoorn et al., 2012). Therapeutically, enhancing ketone-body utilization may compensate for glucose deprivation in metastatic lesions, and combination strategies targeting both glycolysis and ketone-body catabolism may be required to overcome metabolic plasticity.

In summary, the best-supported mechanism is CAF-tumor ketone-body metabolic coupling, in which CAF-derived BHB is utilized by tumor cells to generate acetyl-CoA and support lung colonization. Context-dependent findings include the anti-angiogenic effect of exogenous BHB via HIF-1α suppression, which contrasts with the pro-metastatic effect of enhanced ketone-body utilization. Evidence is strongest for the MDA-MB-231 breast cancer model and CRC colitis-associated cancer model, whereas glioma studies suggest that ketone-body catabolism may be downregulated under hypoxia in certain tumor types. Key unresolved questions include how chronic versus cyclic hypoxia differentially regulates ketone-body utilization across tumor types, and whether compensatory ketolysis limits the efficacy of glycolysis-targeted therapies.

### 2.3 HIF-1α-regulated amino acid metabolic reprogramming drives tumor metastasis

Amino acids are not only building blocks for protein synthesis but also critical regulators of energy metabolism, signal transduction, and redox homeostasis. HIF-1α remodels amino acid metabolism to support tumor proliferation, invasion, and metastasis by enhancing amino acid supply, promoting biosynthesis, and activating recycling pathways. It should be noted that “hypoxia” is a broad term; acute, cyclic, and chronic hypoxia can produce distinct HIF-1α dynamics and amino acid metabolic outputs, and therefore the glutamine, serine/glycine, arginine, and BCAA programs described below should be interpreted in a context-dependent manner.

These pathways can be categorized according to their regulatory relationship with HIF-1α. Direct HIF-1α downstream effects include GLS1 upregulation and BNIP3/BCL2 interacting protein 3 like-mediated autophagy. Upstream regulators include MET-eIF4G1 signaling, whereas NUAKs act as cooperative oncogenic signaling that promotes mTOR/c-MYC-driven Gln metabolic reprogramming. Cooperative oncogenic signaling includes the HIF-1α/c-MYC axis. Bidirectional feedback loops include the SHMT2-HIF-1α axis, in which SHMT2 stabilizes HIF-1α under hypoxia and HIF-1α promotes serine-synthesis gene expression. These distinctions are clarified in each subsection below.

Collectively, these amino acid metabolic programs support local invasion, survival in circulation, colonization, and metastatic outgrowth.

Therapeutically, targeting a single amino acid pathway may induce compensatory reprogramming toward alternative amino acid, glucose, or lipid metabolism; therefore, combination strategies and isotope tracing are required to overcome metabolic plasticity.

To increase amino acid availability, HIF-1α upregulates glutaminase 1 (GLS1), promoting glutamine (Gln) conversion to glutamate (Glu), which enters the TCA cycle or supports the synthesis of other amino acids, thereby providing energy and biosynthetic precursors (Xiang et al., 2019). At the translational level, upstream signaling can regulate HIF-1α synthesis through eukaryotic translation factors and cap-dependent translation. MET signaling activates eIF4G1 Ser1232 phosphorylation through MAPK, thereby regulating HIF-1α translation; MET inhibition decreases eIF4G1 phosphorylation, enhances eIF4G1-eIF4E affinity, and suppresses HIF-1α translation (Glück et al., 2018). In addition to the HIF-1α/c-MYC axis, NUAKs promote mTOR/c-MYC-driven glucose and Gln metabolic reprogramming, supporting breast cancer growth and metastasis (Worral Wilfred Raj and Manoharan, 2024).

HIF-1α also activates recycling mechanisms by inducing BNIP3 and BCL2 interacting protein 3 like. Under hypoxic stress, HIF-1α transcriptionally activates BNIP3, a BH3-only Bcl-2 family protein (Burton and Gibson, 2009). Following mitochondrial localization, BNIP3 promotes autophagy/mitophagy, enabling the degradation and recycling of damaged proteins and organelles to maintain intracellular homeostasis (Sun et al., 2025b; Papandreou et al., 2005).

The following sections describe how HIF-1α regulates Gln/Glu, serine/glycine, arginine, and branched-chain amino acid metabolism to support tumor metastasis.

#### 2.3.1 Glutamine/glutamate

Glutamine metabolism supports local invasion, survival in the circulation, metastatic colonization, and therapy resistance. Tumor cells require Gln to maintain energy homeostasis under hypoxia, supply carbon and nitrogen for macromolecule synthesis, activate EMT-associated programs, and adapt to metabolic stress. Accordingly, many tumors exhibit Gln addiction, characterized by increased transporter expression, enhanced glutaminolysis, and metabolic coordination between HIF-1α and c-MYC. Under hypoxic conditions, Gln may serve as an important energy source and biosynthetic precursor, with Gln-derived carbon supporting TCA-cycle metabolism. The Gln/Glu metabolic response is also influenced by hypoxia mode. Chronic hypoxia may sustain HIF-1α-dependent GLS1 expression and glutaminolysis, whereas cyclic hypoxia may preferentially activate ROS-AMPK-dependent metabolic stress responses; these differences remain to be systematically compared (Xiang et al., 2019; Yun et al., 2023). This program is mainly relevant to local invasion, circulatory survival, metastatic colonization, and therapy resistance.

HIF-1α regulates Gln uptake, catabolism, and downstream signaling. It promotes Glu dehydrogenase (GDH) expression (Jiang et al., 2017), cooperates with c-MYC to increase Gln transporter and GLS1 expression (Lv et al., 2025), and directly induces GLS1 under hypoxia in CRC. GLS1 is required for hypoxia-induced migration, invasion, and metastatic colonization in CRC (Xiang et al., 2019). HIF-1α protein stability is also linked to Gln metabolism, forming a metabolic-transcriptional regulatory loop (Bian et al., 2023). These mechanisms can be categorized into distinct regulatory modes. HIF-1α downstream effects include GLS1 and GDH upregulation. Upstream regulation includes MET-eIF4G1-mediated HIF-1α translation (Glück et al., 2018). Bypass pathways include NUAK-driven mTOR/c-MYC metabolic reprogramming (Worral Wilfred Raj and Manoharan, 2024). Bidirectional feedback is exemplified by the link between HIF-1α stability and glutamine metabolism (Bian et al., 2023).

Mechanistically, HIF-1α and c-MYC increase Gln uptake and Glu production by upregulating Gln transporters and GLS1 in CRC, breast cancer, and HCC (Lv et al., 2025). In lung cancer, HIF-1α-induced GDH promotes Glu-to-α-KG flux and ATP production, helping maintain energy homeostasis under hypoxia and contributing to therapy resistance (Jiang et al., 2017). In CRC, elevated GDH is associated with aggressive and metastatic phenotypes, and has been reported to promote EMT-related signaling (Liu et al., 2015). Curcumol induces HIF-1α degradation, downregulates GLS1, and blocks Gln conversion to Glu and α-KG, thereby suppressing EMT and invasion in CRC cells; HIF-1α or GLS1 overexpression reverses these effects (Bian et al., 2023).

Beyond tumor-cell bioenergetics, enhanced Gln uptake, GLS1-mediated glutaminolysis, and GDH-dependent metabolism may reshape nutrient availability and metabolite composition in the TME, with potential consequences for macrophage polarization and MDSC recruitment (Jiang et al., 2017; Lv et al., 2025). From a therapeutic perspective, inhibition of GLS1 or GDH may trigger compensatory flux rewiring toward fatty acid oxidation or other amino acid pathways to maintain energy supply; isotope tracing and dynamic flux analysis can be applied to characterize these compensatory pathways.

Clinical and experimental studies show that high GLS1 and GDH expression in CRC is associated with EMT, lymph-node metastasis, and distant metastatic risk (Bian et al., 2023; Liu et al., 2015). In lung cancer, GDH expression correlates with hypoxia, chemotherapy resistance, and poor prognosis (Jiang et al., 2017). High expression of Gln transporters in solid tumors is also associated with tumor progression and metastatic potential, while GLS1 inhibitors show anti-metastatic activity in preclinical models (Lv et al., 2025).

In summary, the best-supported mechanism is HIF-1α-dependent upregulation of GLS1 and GDH, which increases glutaminolysis and supports energy homeostasis. Context-dependent findings include tumor-specific reliance on GLS1, GDH, and Gln transporters, as well as upstream (MET-eIF4G1) and bypass (NUAK) regulation. Evidence is strongest for core glutaminolysis enzymes, whereas compensatory pathways remain less validated. Key unresolved questions include how acute, cyclic, and chronic hypoxia differentially reshape glutamine flux and whether combination strategies can overcome metabolic plasticity.

#### 2.3.2 Serine/glycine

Serine and glycine are key one-carbon donors that support local invasion, survival in circulation, distant colonization, and metastatic outgrowth. Tumor cells require these metabolites for nucleotide, glutathione, and phospholipid synthesis, antioxidant defense, adaptation to nutrient deprivation, and epigenetic regulation. Therefore, metastatic tumors often activate the serine-synthesis pathway, increase the expression and activity of enzymes such as phosphoglycerate dehydrogenase (PHGDH), and couple serine metabolism with histone methylation and HIF-1α signaling.

Notably, the serine/glycine metabolic response is influenced by hypoxia mode: chronic hypoxia sustains PHGDH expression and serine synthesis, whereas cyclic hypoxia may modulate the pathway through ROS-AMPK signaling. Spatial hypoxic gradients can further create heterogeneous serine utilization.

This program mainly supports local invasion, distant colonization, and metastatic outgrowth.

HIF-1α regulates serine metabolism through nutrient sensing, transcriptional activation, and positive-feedback mechanisms. During serine/glycine deprivation, ROS-mediated AMPK activation enhances HIF-1α activity, which induces the expression of PHGDH, phosphoserine aminotransferase 1 (PSAT1), and phosphoserine phosphatase. HIF-1α also forms a positive-feedback loop with serine hydroxymethyltransferase 2 (SHMT2), which stabilizes HIF-1α under hypoxia. By altering serine flux, this network affects S-adenosylmethionine (SAM) production and the epigenetic regulation of metastasis-associated genes (Yun et al., 2023; Wang et al., 2023).

These mechanisms can be categorized into distinct regulatory modes. HIF-1α downstream effects include PHGDH, PSAT1, and SHMT2 upregulation. Upstream regulation includes ROS-AMPK-mediated HIF-1α stabilization. Bidirectional feedback is exemplified by the HIF-1α-SHMT2 positive-feedback loop (Yun et al., 2023; Wang et al., 2023).

In glioblastoma, serine/glycine deprivation increases ROS and activates AMPK, thereby enhancing HIF-1α stability and transcriptional activity. Activated HIF-1α upregulates serine-synthesis enzymes, increases intracellular serine levels, supports nucleotide synthesis and glutathione production, and enhances invasiveness and metastatic colonization (Yun et al., 2023). In CRC, Cul4A-DDB1-mediated PHGDH K146 monoubiquitination promotes the interaction of PHGDH with DnaJ heat shock protein family member A1 and favors formation of active PHGDH tetramers. Increased serine synthesis elevates SAM production, which activates SET domain-containing 1A and increases H3K4 trimethylation at the LAMC2 and CYR61 promoters, thereby promoting migration and liver metastasis (Zhang Y. et al., 2021). In addition, SHMT2 stabilizes HIF-1α under hypoxia and regulates downstream VEGF and STAT3 signaling; SHMT2 knockdown suppresses proliferation, colony formation, invasion, and migration in xenograft models (Wang et al., 2023).

PHGDH overexpression in glioblastoma is associated with increased invasiveness, metastatic colonization, and poor prognosis (Yun et al., 2023). In CRC, elevated PHGDH K146 monoubiquitination correlates with liver metastasis and is an independent adverse prognostic factor (Zhang Y. et al., 2021). SHMT2 overexpression in solid tumors is associated with advanced tumor grade, distant metastasis, and shorter survival, whereas targeting serine-synthesis enzymes inhibits metastasis in preclinical models (Wang et al., 2023). From a therapeutic perspective, inhibition of PHGDH or SHMT2 may trigger compensatory flux rewiring toward glycine synthesis or one-carbon metabolism; isotope tracing and dynamic flux analysis can be applied to characterize these compensatory pathways.

In summary, the best-supported mechanism is HIF-1α-dependent upregulation of PHGDH, PSAT1, and SHMT2, which increases serine synthesis and supports nucleotide and glutathione production. Context-dependent findings include tumor-specific reliance on PHGDH and SHMT2, as well as upstream (ROS-AMPK) and bidirectional feedback (HIF-1α-SHMT2) regulation. Evidence is strongest for core serine-synthesis enzymes, whereas compensatory pathways remain less validated. Key unresolved questions include how acute, cyclic, and chronic hypoxia differentially reshape serine flux and whether targeting serine metabolism can overcome metabolic plasticity.

#### 2.3.3 Arginine

During invasion, metastasis, and angiogenesis, tumor cells require sustained HIF transcriptional activity and oncogenic signaling to support metabolic reprogramming and malignant progression. These processes are associated with increased arginine methyltransferase expression, enhanced arginine methylation, and nitric oxide (NO) signaling.

Notably, the arginine metabolic response is influenced by hypoxia mode: chronic hypoxia sustains PRMT1 and CARM1 expression, whereas cyclic hypoxia may modulate arginine methylation through ROS-dependent mechanisms. Spatial hypoxic gradients can further create heterogeneous arginine utilization. This program mainly supports local invasion, angiogenesis, and metastatic colonization.

HIF-1α has been reported to induce protein arginine methyltransferase 1 (PRMT1) expression, while HIF-1α can also interact with coactivator-associated arginine methyltransferase 1 (CARM1) and facilitate its recruitment to target-gene promoters; both mechanisms modulate arginine methylation and may enhance HIF-associated transcriptional activity. In triple-negative breast cancer, CARM1 has been reported to cooperate with HIF-1α-associated transcriptional programs and to promote proliferation, EMT, and stemness (Feng et al., 2024). These mechanisms can be categorized into distinct regulatory modes. HIF-1α downstream effects include PRMT1 induction. HIF-1α-cofactor interaction includes CARM1 recruitment. Bypass pathways include NO-mediated PI3K/AKT and RhoA signaling (Xu et al., 2026).

Under hypoxia, PRMT1 has been reported to methylate hypoxia-inducible factor-2β (HIF-2β; officially designated ARNT2), thereby enhancing HIF binding to DNA and transcriptional activity in breast cancer models (Li W. J. et al., 2025). This mechanism promotes glycolytic gene expression, metabolic reprogramming, and breast cancer progression (Li W. J. et al., 2025). In triple-negative breast cancer, CARM1 interacts with HIF-1α and is recruited to the promoters of CDK4, Cyclin D1, and β-catenin, coordinately promoting proliferation, EMT, and stemness. CARM1 overexpression is associated with poor prognosis (Feng et al., 2024). In addition, NO generated from arginine by NO synthase can activate PI3K/AKT and RhoA signaling, thereby enhancing pancreatic cancer invasiveness (Xu et al., 2026).

Clinical evidence indicates that PRMT1-mediated enhancement of HIF activity contributes to breast cancer progression (Li W. J. et al., 2025), whereas CARM1 overexpression is associated with poor prognosis in triple-negative breast cancer (Feng et al., 2024). NO signaling may further promote pancreatic cancer invasion and metastasis through PI3K/AKT and RhoA pathways (Xu et al., 2026). From a therapeutic perspective, inhibition of PRMT1 or CARM1 may trigger compensatory flux rewiring toward other methyltransferases or NO signaling; isotope tracing and dynamic flux analysis can be applied to characterize these compensatory pathways.

In summary, the best-supported mechanism is HIF-1α-dependent PRMT1 induction and CARM1 interaction, which enhance arginine methylation and HIF transcriptional activity. Context-dependent findings include tumor-specific reliance on PRMT1 and CARM1, as well as bypass (NO) regulation. Evidence is strongest for PRMT1 and CARM1 in breast cancer models, whereas clinical associations remain limited. Key unresolved questions include how acute, cyclic, and chronic hypoxia differentially reshape arginine methylation and whether targeting arginine metabolism can overcome metabolic plasticity.

#### 2.3.4 Branched-chain amino acids

During tumor invasion, metastatic colonization, and cytoskeletal remodeling, tumor cells require nutrient signaling to support protein synthesis, growth, and EMT. They may therefore increase branched-chain amino acid (BCAA) uptake, maintain mechanistic target of rapamycin complex 1 (mTORC1) activation, and redirect BCAA-derived nitrogen through cytosolic branched-chain amino acid transaminase 1 (BCAT1) toward nucleotide synthesis. HIF-1α integrates these nutrient signals into pro-metastatic networks by upregulating the BCAA transporter L-type amino acid transporter 1 (LAT1).

Notably, the BCAA metabolic response is influenced by hypoxia mode: chronic hypoxia sustains LAT1 expression and BCAA uptake, whereas cyclic hypoxia may modulate BCAA metabolism through ROS-dependent mechanisms. Spatial hypoxic gradients can further create heterogeneous BCAA utilization. This program mainly supports local invasion, EMT, and metastatic outgrowth.

In glioblastoma, HIF-1α directly activates LAT1 transcription under hypoxia, increasing leucine and other BCAA uptake. BCAAs subsequently activate mTORC1, induce the EMT transcription factor Snail, and enhance migration and invasion, forming a HIF-1α-LAT1-BCAA-mTORC1-Snail-EMT axis (Zhang B. et al., 2021). In osteosarcoma, BCAAs, particularly leucine, promote growth through LAT1-dependent activation of the AMPK/mTORC1 pathway. Under hypoxia, BCAA metabolism further reinforces HIF-1α-associated mTORC1 signaling, whereas inhibition of BCAA transport or metabolism suppresses mTORC1 activity and pro-metastatic effects (Martin et al., 2020). These mechanisms can be categorized into distinct regulatory modes. HIF-1α downstream effects include LAT1 upregulation. Bypass pathways include BCAT1-mediated UMP synthesis and BCAT2-mediated ammonia release (Ji et al., 2026). Bidirectional feedback is exemplified by the link between BCAA metabolism and HIF-1α-associated mTORC1 signaling (Martin et al., 2020).

In breast cancer, elevated circulating and tissue BCAA levels are accompanied by increased BCAT1 expression. BCAT1 promotes mitochondrial biogenesis and function through mTOR signaling, thereby supporting tumor-cell growth and colony formation independently of AMPK and SIRT1 (Zhang and Han, 2017). In CRC, the subcellular compartmentalization of BCAA metabolism influences metastatic behavior. Cytosolic BCAT1 directs BCAA-derived nitrogen toward uridine monophosphate (UMP) synthesis; UMP binds vimentin and protects it from ubiquitination and degradation, thereby stabilizing vimentin, maintaining EMT and migration, and promoting metastasis. In contrast, mitochondrial BCAT2 releases nitrogen as ammonia, limits UMP synthesis, and suppresses metastasis (Ji et al., 2026).

Clinically, LAT1 overexpression in glioblastoma correlates with HIF-1α activation and invasiveness (Zhang B. et al., 2021). Inhibition of BCAA metabolism weakens mTORC1-driven pro-metastatic effects in osteosarcoma (Martin et al., 2020), while BCAT1 promotes breast cancer mitochondrial function and growth through mTOR signaling (Zhang and Han, 2017). In CRC, the cytosolic BCAT1-UMP-vimentin axis promotes metastasis, whereas mitochondrial BCAT2 restricts this process (Ji et al., 2026). From a therapeutic perspective, inhibition of LAT1 or BCAT1 may trigger compensatory flux rewiring toward other amino acid transporters or metabolic pathways; isotope tracing and dynamic flux analysis can be applied to characterize these compensatory pathways.

In summary, the best-supported mechanism is HIF-1α-dependent LAT1 upregulation and BCAA uptake, which activates mTORC1 and promotes EMT. Context-dependent findings include tumor-specific reliance on LAT1, BCAT1, and BCAT2, as well as bypass (BCAT1-UMP-vimentin) regulation. Evidence is strongest for LAT1 in glioblastoma and BCAT1 in CRC, whereas clinical associations remain limited. Key unresolved questions include how acute, cyclic, and chronic hypoxia differentially reshape BCAA flux and whether targeting BCAA metabolism can overcome metabolic plasticity.

Amino acids function not only as protein precursors but also as regulators of energy metabolism, redox balance, nutrient signaling, and epigenetic control. By remodeling Gln/Glu, serine/glycine, arginine, and BCAA metabolism, HIF-1α supports tumor invasion and colonization through energy supply, antioxidant defense, EMT activation, and metabolic signaling. The major HIF-1α-mediated amino acid metabolic pathways involved in metastasis are summarized in Figure 2.

**FIGURE 2:**
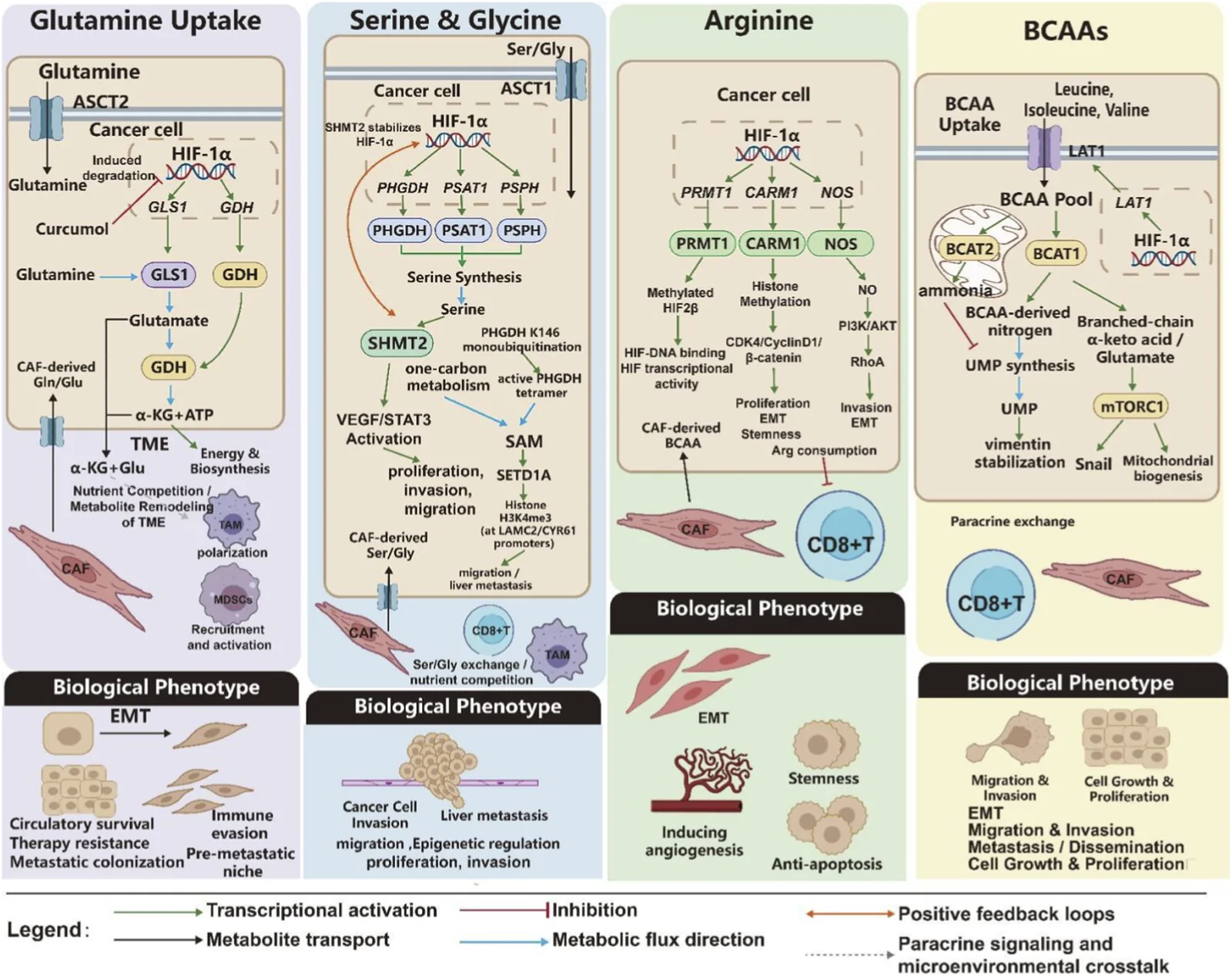
HIF-1α-regulated amino acid metabolic reprogramming drives tumor metastasis. HIF-1α systematically reshapes the amino acid metabolic network to support metastasis. It upregulates GLS1/GDH to promote glutamine catabolism for energy supply. Via the ROS-AMPK axis, it activates *de novo* serine synthesis (PHGDH/PSAT1), while SHMT2 stabilizes HIF-1α under hypoxia. This network supports serine-dependent SAM production, facilitating the epigenetic regulation of metastasis-related genes. It also induces PRMT1 and CARM1 to mediate arginine methylation, enhancing transcriptional activity. Concurrently, HIF-1α upregulates LAT1 to enhance branched-chain amino acid (BCAA) uptake, driving EMT and migration through the mTORC1 pathway and the cytosolic BCAT1-mediated UMP-vimentin axis. These metabolic adaptations ultimately promote EMT, angiogenesis, stemness, and anti-apoptosis, with potential crosstalk within the TME.

### 2.4 HIF-1α-regulated nucleotide metabolic reprogramming drives tumor metastasis

Nucleotides are essential substrates for DNA replication and RNA synthesis in rapidly proliferating tumor cells. Under hypoxia, HIF-1α supports malignant proliferation and metastasis by regulating *de novo* and salvage nucleotide-synthesis pathways to maintain an adequate nucleotide supply. Beyond nucleic acid synthesis, nucleotide metabolic reprogramming also modulates the tumor immune microenvironment through the adenosine pathway.

It should be noted that “hypoxia” is a broad term; acute, cyclic, and chronic hypoxia can produce distinct HIF-1α dynamics and metabolic outputs, and therefore the nucleotide metabolic programs described below should be interpreted in a context-dependent manner.

The following sections discuss xanthine/hypoxanthine metabolism, dNTP synthesis, and the adenosine metabolic axis.

#### 2.4.1 Xanthine/hypoxanthine

During rapid proliferation, local invasion, and angiogenesis, tumor cells require abundant nucleotides for DNA replication while maintaining ROS signaling to stabilize HIF-1α and promote an inflammatory microenvironment. Accordingly, purine catabolism and anabolism can be coordinately reprogrammed: increased xanthine oxidase (XO) and xanthine oxidoreductase (XOR) activity generates ROS that stabilizes HIF-1α and activates inflammatory pathways, whereas HIF-1α enhances *de novo* and salvage purine synthesis to provide nucleotide substrates.

Notably, the purine metabolic response is influenced by hypoxia mode: chronic hypoxia sustains XO/XOR-derived ROS and HIF-1α stabilization, whereas cyclic hypoxia may modulate purine metabolism through intermittent ROS bursts. Spatial hypoxic gradients can further create heterogeneous purine utilization. This program mainly supports local invasion, angiogenesis, and inflammatory microenvironment remodeling.

In chemically induced hypoxic glioma cells, XO-derived ROS inhibits PHD activity, prevents HIF-1α ubiquitination and degradation, and increases its nuclear accumulation. Stabilized HIF-1α subsequently induces GLUT1, PFKFB3, and other glycolytic genes, thereby promoting tumor-cell invasion. XO inhibition with allopurinol or siRNA reduces HIF-1α expression and suppresses these effects (Griguer et al., 2006). In triple-negative breast cancer, hypoxic HIF-1α upregulates key enzymes involved in *de novo* purine synthesis, increasing purine-related metabolites, including xanthine, and supporting nucleotide availability for DNA replication. In addition, HIF-1α translocates to the nucleolus through its nucleolar localization signal, binds ribosomal DNA promoters, and enhances RNA polymerase I activity, rRNA transcription, and ribosome biogenesis. The RNA polymerase I inhibitor BMH-21 suppresses hypoxia-driven invasion and metastasis (Elha et al., 2025).

In EGFR-mutant LUAD, HIF-1α binds the hypoxanthine phosphoribosyltransferase 1 (HPRT1) promoter and activates purine salvage. HPRT1 converts hypoxanthine to inosine monophosphate, supplying nucleotide precursors while enhancing ROS-scavenging capacity and redox homeostasis. HPRT1 inhibition synergizes with gefitinib to induce DNA damage and apoptosis, thereby reducing tumor growth and metastatic potential (Geng et al., 2024).

XO/XOR activity also links purine metabolism to inflammatory microenvironment remodeling. XO/XOR-derived ROS reinforces HIF-1α signaling through PHD inhibition and promotes inflammatory mediator release. Moreover, excessive uric acid can induce oxidative stress, activate the NOD-like receptor family pyrin domain-containing 3 inflammasome, and promote interleukin-1β release, collectively facilitating inflammation-associated malignant progression (Wu et al., 2024; Chen and Meng, 2022).

These mechanisms can be categorized into distinct regulatory modes. HIF-1α downstream effects include upregulation of *de novo* purine synthesis enzymes and HPRT1. Upstream regulation includes XO/XOR-derived ROS stabilizing HIF-1α. Bypass pathways include RNA polymerase I-mediated ribosome biogenesis. Bidirectional feedback is exemplified by the link between purine metabolism and HIF-1α stability (Griguer et al., 2006; Elha et al., 2025; Geng et al., 2024).

From a therapeutic perspective, inhibition of XO/XOR or HPRT1 may trigger compensatory flux rewiring toward alternative purine salvage or pyrimidine synthesis pathways; isotope tracing and dynamic flux analysis can be applied to characterize these compensatory pathways.

Clinical and experimental evidence indicates that XO inhibitors reduce HIF-1α expression and glycolysis-associated invasive phenotypes in glioma (Griguer et al., 2006). High uric acid levels are associated with proliferation and inflammation-related progression in several tumor models (Chen and Meng, 2022). Activation of the HIF-1α/rRNA-biogenesis axis in breast cancer correlates with poor prognosis, whereas RNA polymerase I targeting shows anti-metastatic activity in animal models (Elha et al., 2025). The HIF-1α/HPRT1 axis also promotes proliferation and metastasis in EGFR-mutant LUAD, and HPRT1 inhibition may enhance the efficacy of gefitinib (Geng et al., 2024).

In summary, the best-supported mechanism is HIF-1α-dependent upregulation of purine synthesis and HPRT1-mediated salvage, which supports nucleotide availability and redox homeostasis. Context-dependent findings include tumor-specific reliance on XO/XOR, HPRT1, and RNA polymerase I. Evidence is strongest for HPRT1 in EGFR-mutant LUAD and XO/XOR in glioma, whereas clinical associations remain limited. Key unresolved questions include how acute, cyclic, and chronic hypoxia differentially reshape purine flux and whether targeting purine metabolism can overcome metabolic plasticity.

#### 2.4.2 Deoxyribonucleoside triphosphates (dNTPs)

During rapid cell division, metastatic colonization, and DNA replication stress, tumor cells require an adequate dNTP pool for DNA synthesis while avoiding apoptosis and ferroptosis. Therefore, metastatic tumors may exhibit increased expression of ribonucleotide reductase M2 (RRM2), an expanded dNTP pool, and positive feedback between RRM2 and HIF-1α that supports ferroptosis resistance.

Notably, the dNTP metabolic response is influenced by hypoxia mode: chronic hypoxia sustains RRM2 expression and dNTP pool expansion, whereas cyclic hypoxia may modulate dNTP synthesis through ROS-dependent mechanisms. Spatial hypoxic gradients can further create heterogeneous dNTP utilization. This program mainly supports metastatic colonization, DNA replication stress adaptation, and survival during dormant/reactivation states.

HIF-1α-associated regulation of RRM2 has been reported across multiple tumor types, although direct transcriptional regulation in a pan-cancer context requires further validation. In breast cancer, RRM2 has been associated with EMT-related migration and invasion; whether this phenotype is directly regulated by HIF-1α requires dedicated mechanistic evidence (He et al., 2022). In EGFR-mutant LUAD, mutant EGFR stabilizes HIF-1α, which subsequently activates HPRT1 expression and purine salvage, increasing the supply of purine nucleotides required for DNA replication (Geng et al., 2024).

During intrahepatic cholangiocarcinoma (ICC) colonization, high RRM2 expression simultaneously supports DNA synthesis and ferroptosis resistance. In ICC specifically, HIF-1α directly induces RRM2 (Song et al., 2026); RRM2 overexpression in turn engages HIF-1α-associated signaling, yet a definitive bidirectional RRM2-HIF-1α feedback loop remains to be established. Moreover, Mucin 1 (MUC1) promotes HIF-1α nuclear translocation and stability, thereby enhancing glycolytic gene expression, glucose uptake, and glycolytic flux and redirecting metabolic flux toward *de novo* pyrimidine synthesis (Shukla et al., 2017).

Mechanistically, RRM2 overexpression in ICC expands the dNTP pool, supports malignant proliferation, and promotes resistance to p53-dependent apoptosis. RRM2 also enhances HIF-1α signaling and suppresses ferroptosis through the SLC7A11/GPX4/ACSL4 axis, providing coordinated protection against cell death during tumor progression (Song et al., 2026). HIF-1α-driven glycolysis increases the supply of precursors for pyrimidine synthesis. Enhanced *de novo* pyrimidine synthesis elevates intracellular deoxycytidine triphosphate (dCTP), which competes with the active gemcitabine metabolite 2′,2′-difluorodeoxycytidine triphosphate for DNA incorporation, thereby reducing gemcitabine cytotoxicity and promoting chemoresistance in pancreatic cancer cells (Shukla et al., 2017).

These mechanisms can be categorized into distinct regulatory modes. HIF-1α downstream effects include RRM2 induction and HPRT1 activation. Upstream regulation includes MUC1-mediated HIF-1α stabilization (Shukla et al., 2017). Bypass pathways include EGFR-mutant-mediated HPRT1 activation (Geng et al., 2024). Bidirectional feedback is exemplified by the potential RRM2-HIF-1α positive feedback loop (Song et al., 2026).

From a therapeutic perspective, inhibition of RRM2 or pyrimidine synthesis may trigger compensatory flux rewiring toward purine salvage or alternative nucleotide synthesis pathways; isotope tracing and dynamic flux analysis can be applied to characterize these compensatory pathways.

From a translational perspective, RRM2 and HPRT1 are candidate nodes for disrupting nucleotide supply in hypoxic tumors. RRM2 overexpression promotes EMT in breast cancer and represents a potential therapeutic target (He et al., 2022). The HIF-1α/HPRT1 axis sustains proliferation in EGFR-mutant LUAD (Geng et al., 2024), whereas RRM2 promotes ICC progression through ferroptosis resistance (Song et al., 2026). In pancreatic cancer, combined targeting of HIF-1α or *de novo* pyrimidine synthesis with gemcitabine reduces tumor burden; lower expression of transketolase and CTP synthase, which regulate pyrimidine-synthesis flux, is associated with better outcomes in patients treated with fluoropyrimidine analogs (Shukla et al., 2017). These findings support biomarker-guided combination strategies targeting nucleotide metabolism and provide a rationale for clinical evaluation of nucleotide-directed therapies.

In summary, the best-supported mechanism is HIF-1α-dependent upregulation of RRM2 and coordination with HPRT1-dependent purine salvage, which expands the dNTP pool and supports DNA synthesis and ferroptosis resistance. Context-dependent findings include tumor-specific reliance on RRM2 and HPRT1, as well as upstream MUC1 and bypass EGFR regulation. Evidence is strongest for RRM2 in ICC and HPRT1 in EGFR-mutant LUAD, whereas clinical associations remain limited. Key unresolved questions include how acute, cyclic, and chronic hypoxia differentially reshape dNTP flux and whether targeting nucleotide metabolism can overcome metabolic plasticity.

#### 2.4.3 Adenosine

During immune evasion, HIF-1α-driven nucleotide metabolic reprogramming exerts important immunosuppressive effects through the adenosine pathway. Under hypoxia, increased CD39/CD73-associated extracellular adenosine signaling has been reported in several tumor contexts and may contribute to immunosuppression (Xia et al., 2023). The extent to which this pathway is HIF-1α-dependent is context dependent. CD39 hydrolyzes immunostimulatory extracellular ATP to AMP, whereas CD73 converts AMP to adenosine. Accumulated adenosine acts predominantly through A2A receptors to suppress the effector functions of CD8^+^ T cells and NK cells while enhancing regulatory T-cell (Treg) activity (Xia et al., 2023).

Notably, the adenosine pathway is influenced by hypoxia mode: chronic hypoxia sustains CD39/CD73 expression and adenosine accumulation, whereas cyclic hypoxia may modulate adenosine signaling through intermittent ATP release. Spatial hypoxic gradients can further create heterogeneous adenosine levels. This program mainly supports immune evasion during local invasion and distant colonization.

These mechanisms can be categorized into distinct regulatory modes. HIF-1α downstream effects include CD39/CD73 upregulation (context-dependent). Bypass pathways include adenosine receptor-mediated immunosuppression. Bidirectional feedback is exemplified by the link between hypoxia and adenosine accumulation (Xia et al., 2023). From a therapeutic perspective, inhibition of CD39/CD73 or adenosine receptors may trigger compensatory immunosuppressive pathways; combination strategies with immune checkpoint blockade are required to overcome metabolic plasticity.

This pathway complements lactate-mediated immunosuppression. Lactate creates an acidic microenvironment and epigenetically reprograms immune cells, whereas adenosine provides receptor-mediated inhibitory signaling. Together, these metabolites facilitate immune evasion during invasion and distant colonization (Chen L. et al., 2022; Xia et al., 2023).

In summary, the best-supported mechanism is hypoxia-induced CD39/CD73-mediated adenosine accumulation, which suppresses CD8^+^ T and NK cell effector functions. Context-dependent findings include tumor-specific reliance on CD39/CD73 and adenosine receptors. Evidence is strongest for adenosine-mediated immunosuppression in multiple tumor contexts, whereas direct HIF-1α dependence remains context-dependent. Key unresolved questions include how acute, cyclic, and chronic hypoxia differentially reshape adenosine signaling and whether targeting adenosine metabolism can overcome immune evasion.

Although lipid and nucleotide metabolism have distinct biochemical functions, their hypoxia-driven reprogramming converges to support metastasis. Lipid metabolism supplies membrane components, energy reserves, and signaling lipids, whereas nucleotide metabolism sustains nucleic acid synthesis, replication-stress adaptation, and adenosine-mediated immune evasion. By regulating lipid-associated pathways, including FASN, SCD1, CPT1A, and cholesterol metabolism, together with nucleotide-associated pathways involving HPRT1, RRM2, and the CD39/CD73 axis, HIF-1α integrates biosynthetic capacity with immune adaptation. These mechanisms are summarized in Figure 3.

**FIGURE 3:**
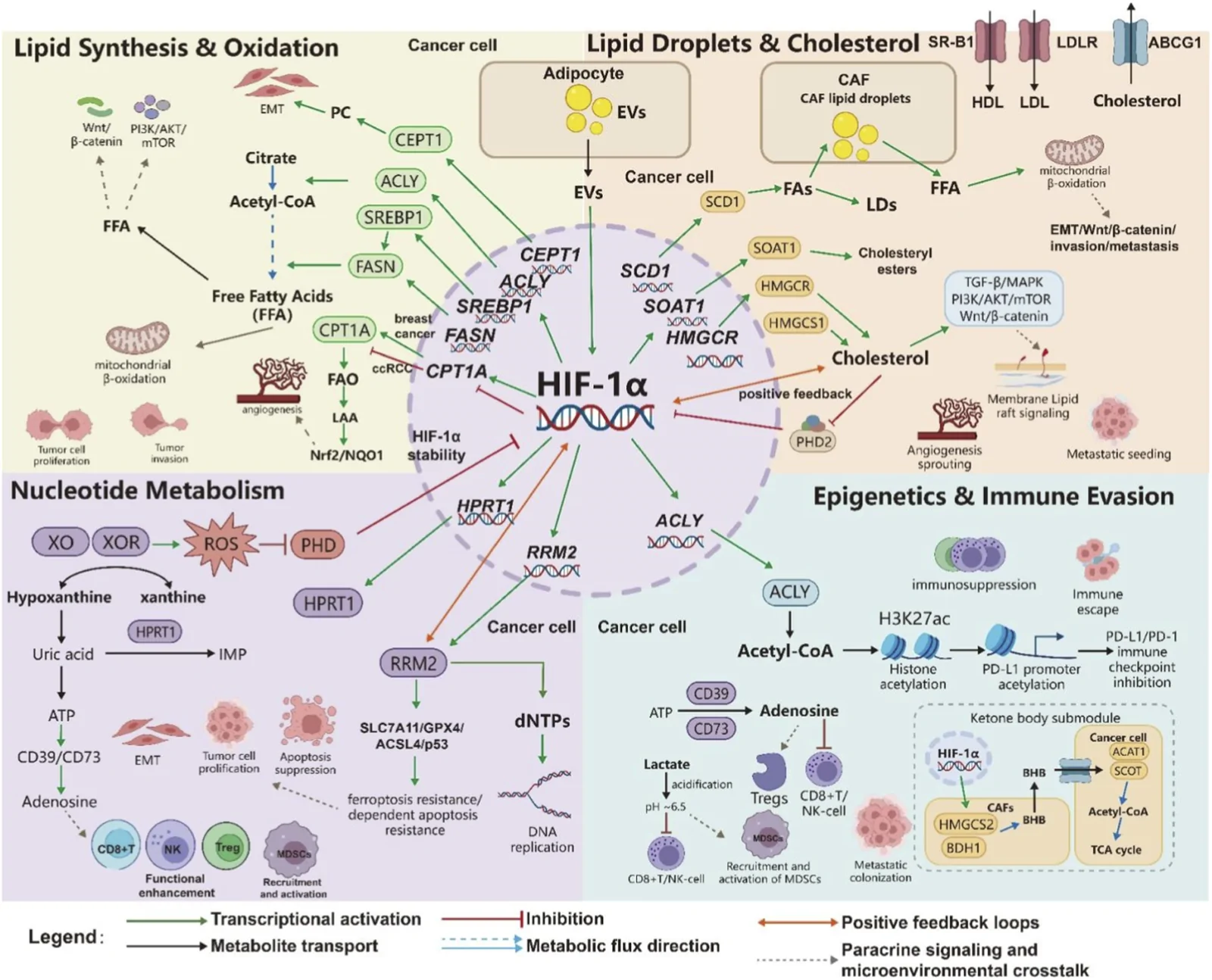
HIF-1α coordinates lipid and nucleotide metabolic reprogramming to drive tumor metastasis. In lipid metabolism, HIF-1α promotes *de novo* fatty acid synthesis and lipid storage through FASN- and SCD1-related pathways, while regulating fatty acid oxidation and cholesterol metabolism (e.g., HMGCR, SOAT1) in a cancer type-dependent manner to support membrane biogenesis, redox adaptation, and signal transduction. In nucleotide metabolism, HIF-1α activates HPRT1-dependent purine salvage and upregulates RRM2 to expand the dNTP pool for DNA replication. In addition, hypoxia-associated CD39/CD73 activity and adenosine signaling contribute to immunosuppression. Notably, these metabolic shifts also induce epigenetic modifications (such as histone acetylation and lactylation) and mediate complex crosstalk with the TME (including CAFs, EVs, and immune cells), coupling biosynthetic support with immune evasion during metastatic progression.

Beyond the pathway-specific mechanisms summarized in Figure 3, HIF-1α-driven metabolic reprogramming extends across glucose, lipid, amino acid, and nucleotide networks. Table 1 summarizes key HIF-1α-associated metabolic programs, their principal molecular mediators, metabolic outputs, and relevance to metastatic progression.

**TABLE 1:** HIF-1α-associated metabolic reprogramming in tumor metastasis: regulators, regulatory relationships, and evidence levels.

Metabolic program	Key regulators	HIF-1α regulatory relationship	Metabolic output	Metastatic stage/organ	Metastatic relevance	Representative cancer types/models	Evidence level	Refs
Glucose uptake	GLUT1, GLUT3	Direct downstream; Indirect/bypass	Increased glucose uptake	Stage: hypoxic survival, local invasion; Organ site: lung	Hypoxic survival; ferroptosis resistance; invasive growth	Esophageal cancer, LUAD, CRC	E1-E2	(Wei et al., 2025; Zhao et al., 2014; Sui et al., 2025; Zhou et al., 2024; Wang et al., 2026)
Glycolytic flux	PKM2, NREP, GPRC5A	Direct downstream; Indirect/bypass	Glycolytic flux; ATP; lactate	Stage: EMT, local invasion; Organ site: lung	EMT; invasion; therapy resistance	Solid tumors, breast cancer, GBM	E1-E2	(Wei et al., 2025; Zhao et al., 2014; Ruan et al., 2024; Alafate et al., 2025)
Lactate generation	LDHA	Direct downstream	Lactate accumulation	Stage: local invasion, immune evasion; Organ site: lung	Acidic TME; immunosuppression; tumor progression	Solid tumors, melanoma	E2; E4	(Wei et al., 2025; Zheng et al., 2024; Zhao et al., 2014)
Pyruvate metabolism	PDK1	Direct downstream	Reduced pyruvate oxidation; increased lactate; reduced TCA flux	Stage: hypoxic adaptation, local invasion; Organ site: not specified (GBM model; no metastatic organ specified in cited refs)	Hypoxic adaptation; invasive growth	GBM	E1-E2	(Kim et al., 2006; Acuña-Pilarte and Koh, 2025; Velpula et al., 2013)
HIF-1α stability and glycolysis	USP11, UBE2S, VHL	Upstream regulator	Glycolytic flux; lactate	Stage: local invasion, metastasis; Organ site: not specified	Glycolysis-dependent migration; metastasis	HCC	E1-E2	(Qiao et al., 2024; Lei and Lee, 2024)
Pentose phosphate pathway	G6PD	Indirect/bypass	Reduced PPP flux; reduced NADPH	Stage: endothelial dysfunction, redox remodeling; Organ site: not specified	Redox remodeling; endothelial dysfunction	Endothelial cell model (non-tumor)	E0	(Yang et al., 2022)
Lactate transport	MCT1, MCT4	MCT1/MCT4: Direct downstream	Lactate uptake; lactate efflux	Stage: metabolic plasticity, metastatic progression; Organ site: not specified	Metabolic plasticity; metastatic progression	Lung cancer	E2; E4	(Li et al., 2025b)
Exosomal glycolytic crosstalk	Exosomal MALAT1	Indirect/bypass	Glucose consumption; ATP; lactate	Stage: metastasis, chemoresistance; Organ site: not specified	Metastasis; chemoresistance	Gastric cancer	E2	(Wang et al., 2024a)
Lactate-mediated feedback signaling	ABCF1	Bidirectional feedback	Lactate accumulation; protein lactylation	Stage: local invasion; Organ site: lung	HIF-1α amplification; lung metastasis	HCC	E2; E4	(Hong et al., 2025)
Lactate-mediated mitophagy	BNIP3, histone lysine lactylation	Direct downstream (BNIP3); Indirect/bypass (histone lactylation)	Mitophagy; histone lactylation	Stage: tumor growth, local invasion; Organ site: not specified	Proliferation; invasion	Glioma	E1-E2	(Dong et al., 2025)
Lactate-mediated ferroptosis resistance	SLC1A1	Direct downstream	Cystine uptake; glutathione synthesis	Stage: metastatic survival, ferroptosis resistance; Organ site: not specified	Metastatic survival; ferroptosis resistance	Solid tumors	E1-E2	(Yang et al., 2023)
Succinate metabolism	P4HA1, SDHB, SDHC	Bidirectional feedback; Indirect/bypass	Succinate accumulation	Stage: ECM remodeling, EMT, local invasion; Organ site: liver	ECM remodeling; EMT; invasion	GBM, breast cancer, CRC	E2; E4	(Yang et al., 2024; Ding et al., 2024)
Citrate and acetyl-CoA metabolism	ACLY, SLC25A1	Direct downstream; Indirect/bypass	Cytosolic citrate; acetyl-CoA	Stage: lipogenesis, stress survival, metastatic colonization; Organ site: peritoneum, lung	Lipogenesis; stress survival; metastatic colonization	Gastric cancer, ccRCC, HCC, CRC	E2; E4	(Zhang et al., 2025b; Yang et al., 2021; Ma et al., 2023; Hu et al., 2025)
α-KG metabolism	IDH2	Indirect/bypass	Reduced α-KG	Stage: HIF-1α stabilization, local invasion; Organ site: lymph node	HIF-1α stabilization; invasion	CRC	E2; E4	(Liu et al., 2024a)
Fatty acid synthesis	FASN	Direct downstream; Indirect/bypass (context-dependent)	Free fatty acids	Stage: redox defense, migratory growth, metastatic growth; Organ site: not specified	Redox defense; migratory growth	CRC, solid tumors	E1-E2	(Lee et al., 2015; Guo et al., 2021; Ni et al., 2017; Wang et al., 2026)
Fatty acid oxidation	CPT1A, ACSL3	Direct downstream; Indirect/bypass (context-dependent)	Reduced FAO in ccRCC; increased FAO in breast cancer; ATP and NADPH support	Stage: EMT, angiogenesis, distant colonization; Organ site: not specified	EMT; angiogenesis; metastatic colonization	ccRCC, breast cancer, CRC	E2	(Du et al., 2017; Quan et al., 2022; Ma et al., 2025)
Fatty acid desaturation	SCD1	Direct downstream	Monounsaturated fatty acids; lipid droplets	Stage: CAF metabolic crosstalk, EMT, local invasion; Organ site: not specified	CAF metabolic crosstalk; EMT; invasion	Lung cancer	E2	(Zhang et al., 2022)
Acetyl-CoA homeostasis	MCAD, LCAD, ACOT12	Indirect/bypass	Reduced FAO-derived acetyl-CoA; acetyl-CoA accumulation	Stage: oxidative stress adaptation, epigenetic EMT; Organ site: distant metastasis	Oxidative stress adaptation; epigenetic EMT	HCC	E2; E4	(Huang et al., 2014; Lu et al., 2019)
Acetate crosstalk	ALDH2 in TAMs	Indirect/bypass (context-dependent)	Acetate; acetyl-CoA	Stage: TAM-mediated EMT; Organ site: distant metastasis	TAM-mediated EMT; distant metastasis	HCC	E2	(Shen et al., 2025)
Cholesterol metabolism	SR-B1, SREBP2, HMGCS1, SOAT1	Bidirectional feedback; Indirect/bypass	Cholesterol; cholesterol esters	Stage: quiescent-cell survival, EMT, local invasion; Organ site: lung	Quiescent-cell survival; EMT; invasion	Melanoma, HCC	E2; E4	(Eckel et al., 2025; Liu et al., 2024b; Yang et al., 2025; Fu et al., 2024)
Phospholipid metabolism	CEPT1, FADS2	Direct downstream; Indirect/bypass	Phosphatidylcholine; membrane PUFA	Stage: membrane plasticity, confined migration, EMT; Organ site: not specified	Membrane plasticity; confined migration; EMT	Gastric cancer, multiple tumors	E2	(Zhou et al., 2025; Chen et al., 2025)
Ketone body metabolism	OXCT1	Indirect/bypass (context-dependent)	Ketolysis; acetyl-CoA	Stage: tumor growth, metastatic colonization; Organ site: not specified	Tumor growth; metastatic colonization	HCC	E2	(Guo et al., 2025)
Glutamine metabolism	GLS1, GDH, NUAK1, NUAK2	Direct downstream; Indirect/bypass	Glu; α-KG; ATP	Stage: redox support, EMT, therapy resistance, metastatic colonization; Organ site: not specified	Redox support; EMT; therapy resistance	CRC, breast cancer, HCC, lung cancer	E2	(Xiang et al., 2019; Worral Wilfred Raj and Manoharan, 2024; Jiang et al., 2017; Lv et al., 2025)
Serine and glycine metabolism	PHGDH, SHMT2	Direct downstream; Bidirectional feedback	Serine; SAM; one-carbon units	Stage: biosynthetic support, epigenetic plasticity, colonization; Organ site: liver	Biosynthetic support; epigenetic plasticity; colonization	GBM, CRC, gastric cancer	E2; E4	(Yun et al., 2023; Wang et al., 2023; Zhang et al., 2021a)
Arginine signaling	PRMT1, CARM1	Indirect/bypass (HIF-cofactor interaction)	HIF-2β methylation; oncogenic transcription	Stage: stemness, EMT, malignant progression; Organ site: not specified	Stemness; EMT; malignant progression	Breast cancer, TNBC	E2; E4	(Feng et al., 2024; Li et al., 2025c)
BCAA metabolism	LAT1, BCAT1, BCAT2	Direct downstream; Indirect/bypass	BCAA uptake; UMP; ammonia	Stage: dissemination, EMT, metastasis; Organ site: not specified	BCAT1 promotes dissemination; BCAT2 restrains dissemination	GBM, osteosarcoma, CRC	E2; E4	(Zhang et al., 2021b; Martin et al., 2020; Ji et al., 2026)
Purine metabolism	XO, XOR, HPRT1	Upstream regulator; Direct downstream	ROS; uric acid; hypoxanthine; inosine monophosphate	Stage: HIF-1α stabilization, redox survival, inflammatory progression, therapy resistance; Organ site: not specified	HIF-1α stabilization; redox survival; inflammatory progression	Glioma, EGFR-mutant LUAD	E1-E2	(Griguer et al., 2006; Geng et al., 2024)
Nucleotide synthesis	RRM2, pyrimidine synthesis enzymes	Direct downstream (RRM2 in ICC); Indirect/bypass (pyrimidine synthesis)	dNTPs; dCTP	Stage: replication support, ferroptosis resistance, drug resistance, metastatic colonization; Organ site: not specified	Replication support; ferroptosis resistance; drug resistance	Breast cancer, ICC, pancreatic cancer	E1-E2	(He et al., 2022; Song et al., 2026; Shukla et al., 2017)
Adenosine signaling	CD39, CD73	Direct downstream (CD39/CD73)	Adenosine	Stage: immune evasion, metastatic colonization; Organ site: not specified	Immune evasion; metastatic colonization	Multiple tumors	E0-E1	(Xia et al., 2023)
ROS signaling	Mitochondrial complex III, SERPINE1	Upstream regulator; Direct downstream	Mitochondrial ROS; SERPINE1	Stage: local invasion, migration, vasculogenic mimicry; Organ site: lung	Invasion; migration; vasculogenic mimicry	Melanoma, GBM	E2	(Comito et al., 2011; Zhang et al., 2023)
SAM metabolism	PCK1	Indirect/bypass (anti-metastatic protective context)	SAM; H3K9me3	Stage: metastasis suppression; Organ site: not specified	Metastasis suppression	HCC	E2; E4	(Gou et al., 2023)

Importantly, the consequences of metabolic rewiring extend beyond altered enzymatic flux and biomass production. Metabolites generated or accumulated during this process, including ROS, lactate, acetyl-CoA, α-KG, ketone bodies, and methyl donors, can function as signaling molecules that regulate tumor-cell plasticity, epigenetic states, and metastatic-niche formation. The following section therefore focuses on metabolite-mediated regulatory mechanisms.

### 2.5 Metabolite-mediated signaling, epigenetic remodeling, and tumor microenvironment adaptation in metastasis

The products of tumor metabolic reprogramming are not merely energy sources and biosynthetic building blocks; they also function as signaling and regulatory molecules that promote metastasis by reshaping epigenetic states and generating immunosuppressive microenvironments. By coordinating metabolite production, epigenetic modification, inflammatory signaling, and metabolic crosstalk with stromal and immune cells, HIF-1α acts as a central driver of these processes. It should be noted that “hypoxia” is a broad term; acute, cyclic, and chronic hypoxia can produce distinct HIF-1α dynamics and metabolic outputs, and therefore the metabolite-mediated signaling and epigenetic remodeling described below should be interpreted in a context-dependent manner.

#### 2.5.1 ROS

During the initiation of metastasis and subsequent distant colonization, tumor cells have distinct, stage-specific requirements for oxidative stress. At the invasion-initiation stage, they use mitochondrial ROS as signaling molecules to stabilize HIF-1α and activate metastatic programs. During colonization, they reduce cytotoxic ROS through HIF-1α-mediated metabolic reprogramming to limit oxidative damage and promote survival.

Notably, ROS signaling is influenced by hypoxia mode: acute/cyclic hypoxia promotes ROS bursts that stabilize HIF-1α, whereas chronic hypoxia may favor antioxidant adaptation. Spatial hypoxic gradients can further create heterogeneous ROS levels. This program is most relevant to local invasion, extravasation, and distant colonization.

HIF-1α is both a responder to ROS signals and a regulator of intracellular ROS levels. During metastatic initiation, hypoxia promotes ROS production from mitochondrial respiratory-chain complex III. ROS inhibit PHD activity and stabilize HIF-1α, which subsequently activates the MET proto-oncogene and drives melanoma invasion, extracellular-matrix dissemination, three-dimensional matrix invasion, metastatic-colony growth, and vasculogenic mimicry (Comito et al., 2011). In glioblastoma, hypoxia-induced ROS similarly stabilizes HIF-1α and promotes migration and invasion through EMT. HIF-1α also binds the SERPINE1 promoter and increases SERPINE1 expression, further enhancing migration and invasion (Zhang et al., 2023).

During pulmonary metastatic colonization, cancer cells transiently activate HIF-1α in a ROS-dependent but hypoxia-independent manner after extravasation. HIF-1-mediated metabolic reprogramming subsequently decreases cytotoxic ROS levels, thereby promoting survival and lung colonization (Zhao et al., 2014).

These mechanisms can be categorized into distinct regulatory modes. HIF-1α downstream effects include MET and SERPINE1 activation. Upstream regulation includes ROS-mediated HIF-1α stabilization. Bidirectional feedback is exemplified by the link between ROS and HIF-1α. From a therapeutic perspective, targeting ROS signaling may trigger compensatory antioxidant pathways; isotope tracing and dynamic flux analysis can be applied to characterize these compensatory pathways.

Available evidence indicates that hypoxia-driven mitochondrial ROS is a major driver of melanoma invasion, with the HIF-1α/MET axis serving as a central regulator (Comito et al., 2011). The ROS-HIF-1α-SERPINE1 axis contributes to glioblastoma migration and invasion (Zhang et al., 2023), whereas HIF-1-mediated antioxidant protection is critical for successful pulmonary colonization (Zhao et al., 2014).

In summary, the best-supported mechanism is ROS-mediated HIF-1α stabilization and downstream MET/SERPINE1 activation, which promotes invasion and colonization. Context-dependent findings include stage-specific ROS requirements and tumor-specific reliance on MET and SERPINE1. Evidence is strongest for melanoma and glioblastoma models, whereas clinical associations remain limited. Key unresolved questions include how acute, cyclic, and chronic hypoxia differentially reshape ROS signaling and whether targeting ROS can overcome metabolic plasticity.

The metabolic dependencies described above provide potential anti-metastatic vulnerabilities. Because HIF-1α-driven metabolic reprogramming supports survival, invasion, immune evasion, and distant colonization through interconnected pathways, pharmacological inhibition of key metabolic enzymes or transporters may disrupt several malignant processes simultaneously. Rational combinations of metabolic inhibitors with chemotherapy, targeted therapy, or immune checkpoint blockade may further help overcome metabolic plasticity and improve therapeutic efficacy.

These therapeutic opportunities also depend on how metabolic intermediates influence gene expression. Beyond their roles in bioenergetics, metabolites such as acetyl-CoA, lactate, and α-KG can modify chromatin and immune-cell states, linking HIF-1α-driven flux to epigenetic plasticity. Considering this metabolite-mediated layer may therefore refine combination design, reveal vulnerabilities not captured by targeting metabolic enzymes alone, and improve the durability of anti-metastatic therapies by helping overcome compensatory metabolic rewiring in metastatic tumors.

Although direct inhibition of metabolic enzymes and transporters is an important therapeutic strategy, targeting metabolic flux alone may not fully suppress metastasis. Metabolic intermediates also exert non-canonical regulatory effects as substrates, cofactors, or inhibitors of epigenetic-modifying enzymes. These metabolite-dependent epigenetic effects provide an additional layer through which HIF-1α-driven metabolic reprogramming promotes tumor progression and metastasis.

#### 2.5.2 Metabolite-mediated epigenetic remodeling drives metastasis

By serving as substrates, cofactors, or inhibitors of chromatin-modifying enzymes, metabolites directly regulate histone and DNA modifications, thereby affecting the expression of metastasis-related genes.

Notably, metabolite-mediated epigenetic remodeling is influenced by hypoxia mode: chronic hypoxia sustains metabolite availability for epigenetic modifications, whereas cyclic hypoxia may modulate epigenetic enzymes through ROS-dependent mechanisms. These mechanisms are most relevant to local invasion, colonization, and metastatic outgrowth.

These mechanisms can be categorized into distinct regulatory modes. HIF-1α downstream effects include acetyl-CoA and lactate production. SAM synthesis represents a context-dependent, HIF-1α-independent protective metabolic-epigenetic axis: PCK1 supports SAM biosynthesis, and SAM-mediated H3K9me3 enrichment represses metastasis-promoting genes, thereby limiting HCC metastasis. Bidirectional feedback is exemplified by the link between metabolites and HIF-1α.

In summary, the best-supported mechanism is metabolite-driven epigenetic remodeling through acetyl-CoA, lactate, and SAM. Context-dependent findings include tumor-specific reliance on different epigenetic modifications. Evidence is strongest for lactate and acetyl-CoA in multiple tumors, whereas SAM-mediated repression is context-dependent. Key unresolved questions include how acute, cyclic, and chronic hypoxia differentially reshape epigenetic states and whether targeting epigenetic-metabolic axes can overcome plasticity.

##### 2.5.2.1 SAM

During invasion and colonization, histone-methylation remodeling influences the activation or silencing of metastasis-related genes. Tumor cells may reduce SAM synthesis and the enrichment of the repressive histone mark H3K9me3 at promoters of metastasis-promoting genes, resulting in epigenetic derepression and enhanced metastatic capacity. In contrast to HIF-1α-associated acetyl-CoA- and lactate-mediated activating modifications, SAM, which an integration node of one-carbon and serine metabolism, can act as a metastasis barrier in specific contexts by supporting repressive histone methylation.

Notably, SAM-mediated epigenetic remodeling is influenced by hypoxia mode: chronic hypoxia may reduce SAM availability, whereas cyclic hypoxia may modulate SAM synthesis through ROS-dependent mechanisms. This program is most relevant to local invasion and colonization.

In HCC, low expression of the gluconeogenic enzyme PCK1 is an important upstream event. PCK1 supports SAM biosynthesis, and SAM supplies methyl groups for H3K9me3 modification. Increased H3K9me3 enrichment at the promoters of EMT- and invasion-associated genes represses their transcription, thereby limiting migration and invasion. PCK1 overexpression increases H3K9me3 and suppresses metastasis, whereas PCK1 knockdown reduces this repressive modification, derepresses metastasis-promoting genes, and enhances metastatic capacity (Gou et al., 2023).

These mechanisms can be categorized into distinct regulatory modes. Upstream regulation includes PCK1-mediated SAM synthesis. Bypass pathways include alternative methylation pathways. Bidirectional feedback is not firmly established for SAM. From a therapeutic perspective, targeting SAM metabolism may trigger compensatory methylation pathways; combination strategies are required.

Clinically, low PCK1 expression in HCC correlates with poor prognosis and increased metastatic potential. The PCK1-SAM-H3K9me3 axis therefore represents a metabolic-epigenetic pathway that suppresses HCC metastasis (Gou et al., 2023). This finding highlights that metabolite-mediated epigenetic regulation is not uniformly pro-metastatic; its effects depend on the type of modification, target-gene function, and tumor context. These differences should be considered when developing therapies targeting metabolic-epigenetic axes.

In summary, the best-supported mechanism is PCK1-SAM-H3K9me3-mediated repression of metastasis-promoting genes. Context-dependent findings include tumor-specific reliance on SAM metabolism. Evidence is strongest for HCC, whereas clinical associations remain limited. Key unresolved questions include how acute, cyclic, and chronic hypoxia differentially reshape SAM availability and whether targeting SAM metabolism can suppress metastasis.

Collectively, HIF-1α-driven metabolic reprogramming functions not only as a source of energy and biosynthetic substrates but also as a signaling network that shapes the epigenetic and immunological landscape of metastasis. Lactate, acetyl-CoA, fatty acids, ketone bodies, and α-KG can regulate histone modifications, immune-cell polarization, and metastatic-niche formation in a context-dependent manner. Table 2 summarizes these metabolite-mediated mechanisms and their relationships with HIF-1α signaling, tumor-microenvironment remodeling, and metastatic progression.

**TABLE 2:** Metabolite-driven epigenetic and microenvironmental programs linked to HIF-1α signaling in metastasis.

Metabolic axis or epigenetic program	Core mechanism	Main TME cell type or metastatic niche	HIF link	Biological consequence	Metastatic implication or stage	Cancer type or model	Refs
Lactate	ABCF1 K430 lactylation; KDM3A activation; reduced H3K9me2	HCC cells; lung metastatic niche	Metabolite-to-HIF feedback	HIF-1α amplification; pro-metastatic signaling	Local invasion; lung colonization	HCC	(Hong et al., 2025)
Lactate	ACSS2-derived lactyl-CoA; KAT2A-mediated histone lactylation	CD8^+^ T cells; NK cells; MDSCs; liver and lung niches	HIF-associated paracrine TME crosstalk; metabolite-to-HIF feedback	PD-L1 induction; impaired cytotoxicity; MDSC recruitment; M2 polarization	Immune evasion; metastatic dissemination	NSCLC; pancreatic cancer; multiple solid tumors	(Chen et al., 2022b; Xia et al., 2023; Zhang et al., 2024d; Wang et al., 2024d; Zhu et al., 2025)
Lactate and BNIP3	BNIP3-dependent mitophagy; histone lactylation	Glioma cells	HIF-1α-dependent indirect regulation	Metabolic reprogramming; pro-invasive gene expression	Tumor growth; local invasion	Glioma	(Dong et al., 2025)
Acetyl-CoA	Nuclear acetyl-CoA; p300-mediated H3K27ac at CD274	Melanoma cells; T-cell surveillance niche	HIF-1α-associated metabolic regulation	PD-L1 induction; reduced T-cell activation	Immune evasion	Melanoma	(Wang et al., 2024b)
TAM-derived acetate and acetyl-CoA	TAM acetate production; histone acetylation; EMT-gene activation	TAMs; HCC cells	HIF-associated paracrine TME crosstalk	Acetate reservoir; EMT activation; immune suppression	Immune evasion; distant metastasis	HCC	(Shen et al., 2025)
Acetyl-CoA	ACOT12 repression; H3K27ac at Snail and Twist promoters	HCC cells	HIF-1α-dependent indirect regulation	EMT-transcription-factor activation	Local invasion; metastasis	HCC	(Lu et al., 2019)
Acetyl-CoA	Histone H3 acetylation; CXCL1 induction	Tumor-associated neutrophils; NETs	HIF-1α-dependent indirect regulation	TAN recruitment; NET formation; inflammatory niche	Metastatic niche formation	HCC	(Pan et al., 2024b)
Free fatty acids	FASN and SCD1 activation; FFA accumulation; Wnt signaling	Tumor cells; CAFs; lipid-rich TME	Direct HIF-1α targets	Lipid support; β-catenin activation; invasive capacity	Local growth; metastatic outgrowth	CRC; lung cancer	(Ni et al., 2017; Zhang et al., 2022)
Oleic acid	PPARγ and FABP4 feedback; fatty-acid uptake and oxidation	Lymph-node metastatic niche	HIF-associated paracrine TME crosstalk	Lipid adaptation; immunosuppression; therapy resistance	Lymph-node colonization	Cholangiocarcinoma	(Zhang et al., 2024c)
Linoleic acid	LINC01116, EWSR1, PPARA, and FABP1 signaling	T cells; paracrine TME	HIF-associated paracrine TME crosstalk	Essential-fatty-acid competition; impaired T-cell function	Immune evasion; tumor progression	HCC	(Ma et al., 2024c)
*De novo* lipogenesis and lipid droplets	FASN-dependent lipogenesis; lipid storage; fatty-acid oxidation	Metastatic tumor cells	Direct HIF-1α target	Anoikis resistance; migratory fitness	Migration; distant colonization	Breast cancer	(Andolino et al., 2025)
Fatty-acid synthesis	METTL3 and TCF7L2 signaling; fatty-acid remodeling; histone acetylation	Clear cell renal cell carcinoma cells	HIF-family parallel axis	EMT; epigenetic remodeling; metabolic reprogramming	EMT; metastasis	Clear cell renal cell carcinoma	(Shi et al., 2024c)
Ketone bodies, BHB	Reduced HIF-1α nuclear activity; reduced VEGFA transcription	Endothelial cells; CRC	HIF-independent counter-regulatory mechanism	Reduced angiogenesis; reduced tumor progression	Anti-metastatic counter-regulation; reduced lung metastasis	CRC	(Huang et al., 2024)
Ketone bodies, BHB and acetoacetate	CAF ketogenesis; ketone-body secretion; tumor-cell ketolysis	CAFs; breast cancer cells	HIF-associated paracrine TME crosstalk	Acetyl-CoA regeneration; increased invasive fitness	Invasion; lung colonization	Breast cancer	(Martinez-Outschoorn et al., 2012)
α-KG	Low α-KG; PHD inhibition; JMJD inhibition	Breast cancer metastatic niche	Metabolite-to-HIF feedback	HIF-1α stabilization; EMT; pro-metastatic epigenetic program	Local invasion; distant metastasis	Breast cancer	(Xiong et al., 2023)
ASH1L, HIF-1α, and IGF-2	ASH1L-HIF-1α cooperation; IGF-2 activation; TAM oxidative and lipid remodeling	Bone metastatic niche; lipid-associated TAMs	HIF-associated epigenetic cofactor interaction	TAM oxidative phosphorylation; lipid-associated differentiation	Bone colonization; metastatic outgrowth	Prostate cancer bone metastasis	(Meng et al., 2025)

Taken together, the evidence summarized above supports a systems-level model in which hypoxia-induced HIF-1α activation integrates metabolic flux remodeling with epigenetic regulation and TME reprogramming. This multidimensional regulatory network explains why metastatic tumors exhibit substantial metabolic plasticity and also provides a rationale for developing combination strategies that simultaneously target metabolism, immune evasion, and metastatic adaptation.

Importantly, the spatial and temporal heterogeneity of hypoxia, including acute, cyclic, and chronic hypoxia, should be considered when designing such combination strategies.

## 3 Therapeutic targeting of HIF-1α-associated metabolic vulnerabilities

Building on the mechanistic landscape described in Section 2, hypoxia-driven HIF-1α signaling provides multiple therapeutic entry points. This section integrates Tables 3, 4; evidence is graded using the E0–E6 hierarchy. Table 3 covers direct HIF-1α-targeting agents and downstream metabolic enzyme/transporter inhibitors, including tumor context, treatment modality, trial status, evidence source/level, and whether anti-metastatic benefit has been proven. Table 4 covers combination strategies, resistance mechanisms, toxicity, candidate biomarkers, delivery approaches, and evidence status.

**TABLE 3:** HIF-1α-associated metabolic interventions: clinical status, evidence, and anti-metastatic endpoints.

Category	Target	Agent	Tumor	Treatment	Trial	Source	Level	Metastasis endpoint (proven?)	Refs
Direct HIF-1α	HIF-1α	EZN-2968	Refractory advanced solid tumors	Monotherapy	NCT01120288; Phase I; Completed	Cell line; animal; early clinical	E5	Not assessed; no proven anti-metastatic benefit	(Fallah and Rini, 2019)
Direct HIF-1α	HIF-1α	PX-478	Advanced solid tumors or lymphoma	Monotherapy	NCT00522652; Phase I; Completed	Cell line; animal; early clinical	E5	Not assessed; no proven anti-metastatic benefit	(Fallah and Rini, 2019)
Indirect/nanomedicine	HIF-1α (indirect)	CRLX101	Ovarian/tubal/peritoneal cancer	+ bevacizumab	NCT01652079; Phase II; Completed	Animal; early clinical	E5	PFS at 6 months; no proven anti-metastatic benefit	(Fallah and Rini, 2019)
Direct HIF-1α	HIF-1α	HIF-1α siRNA/CRISPR	Multiple solid tumors	Nanoparticle delivery	Not available from cited references	Cell line; animal	E1	Preclinical metastasis; preclinical only	(Fallah and Rini, 2019)
Downstream	PDK1	BX-795	Glioblastoma	+ EGFR inhibition (gefitinib)	Preclinical; NA	Cell line; animal	E1	Preclinical growth; mechanistic only	(Velpula et al., 2013)
Downstream	LDHA	FX-11; GSK2837808A	Lung cancer; CRC; HCC	Chemotherapy; monotherapy	Preclinical; NA	Cell line; animal metastasis model	E2	Invasion; metastasis in preclinical models; preclinical only	(Zheng et al., 2024; Yang et al., 2023; Hong et al., 2025)
Downstream	GLUT1	WZB117; BAY-876	Esophageal cancer; LUAD	+ EGFR inhibition	Preclinical; NA	Cell line; animal	E1	Preclinical growth; preclinical only	(Chen et al., 2022a; Sui et al., 2025; Zhou et al., 2024)
Downstream	MCT1	AZD3965	Advanced solid tumors; lymphoma	Monotherapy	NCT01791595; Phase I; Completed	Preclinical; early clinical	E5	Not assessed; no proven anti-metastatic benefit	(Li et al., 2025b; Chen et al., 2022b; Xia et al., 2023)
Downstream	MCT4	MCT4 inhibition (genetic/pharmacologic)	Lung cancer; HCC	+ PD-1/PD-L1	Preclinical; NA	Cell line; animal	E1	Preclinical lactate efflux; preclinical only	(Li et al., 2025b; Chen et al., 2022b; Xia et al., 2023)
Downstream	IDH2	Enasidenib	IDH2-mutant AML	Monotherapy	NCT02577406; Phase III; Completed	Approved clinical (other indication)	E6*	Not applicable; approval not anti-metastatic	(Liu et al., 2024a; Xiong et al., 2023)
Downstream	IDH2	Enasidenib; AGI-6780	CRC	Monotherapy	Preclinical; NA	Cell line; animal	E2	Preclinical metastasis; preclinical only	(Liu et al., 2024a; Xiong et al., 2023)
Downstream	FASN	TVB-2640	CRC; solid tumors	Monotherapy	NCT02223247; Phase I; Completed	Preclinical; early clinical	E5	Not assessed; no proven anti-metastatic benefit	(Lee et al., 2015; Wang et al., 2026)
Downstream	FASN	TVB-2640	HER2+ metastatic breast cancer	+ trastuzumab/paclitaxel	NCT03179904; Phase II; Ongoing	Preclinical; early clinical	E5	Not assessed; no proven anti-metastatic benefit	(Lee et al., 2015; Wang et al., 2026)
Downstream	SCD1	A939572	Lung cancer; CRC	Chemotherapy; monotherapy	Preclinical; NA	Cell line; animal	E2	Preclinical EMT; preclinical only	(Wang et al., 2026; Zhang et al., 2022)
Downstream	CPT1A/FAO	Etomoxir	ccRCC; breast cancer	Monotherapy	Preclinical; NA	Cell line; animal	E1	Mechanistic; mechanistic only	(Du et al., 2017; Ma et al., 2025)
Downstream	ACLY/USP13	ACLY inhibition; USP13 silencing	Melanoma; HCC	+ PD-1/PD-L1	Preclinical; NA	Cell line; animal	E1	Preclinical immune evasion; mechanistic only	(Hu et al., 2025; Wang et al., 2024b)
Downstream	GLS1	CB-839 (telaglenastat)	solid tumors; NSCLC	Monotherapy; + sapanisertib	NCT04250545; Phase I/Ib; Ongoing	Preclinical; early clinical	E5	Not assessed; no proven anti-metastatic benefit	(Lv et al., 2025)
Downstream	GLS1	CB-839 (telaglenastat)	NF1-mutant solid tumors	Monotherapy	NCT03872427; Phase II; Ongoing	Preclinical; early clinical	E5	Not assessed; no proven anti-metastatic benefit	(Lv et al., 2025)
Downstream	GLS1	Curcumol	CRC	Monotherapy	Preclinical; NA	Cell line; animal	E2	Preclinical EMT; preclinical only	(Bian et al., 2023)
Downstream	PHGDH	PHGDH inhibitors (e.g., NCT-503, BI-4916)	Glioblastoma; CRC	Monotherapy	Preclinical; NA	Cell line; animal	E2	Preclinical colonization; preclinical only	(Yun et al., 2023; Zhang et al., 2021a)
Downstream	LAT1	JPH203 (nanvuranlat)	solid tumors	Monotherapy	UMIN000016546; Phase I; Completed	Preclinical; early clinical	E5	Not assessed; no proven anti-metastatic benefit	(Zhang et al., 2021b; Martin et al., 2020)
Downstream	LAT1	JPH203 (nanvuranlat)	Biliary tract cancer	Monotherapy	UMIN000034080; Phase II; Completed	Preclinical; early clinical	E5	PFS improvement; no proven anti-metastatic benefit	(Zhang et al., 2021b; Martin et al., 2020)
Downstream	RRM2	Berberine	Breast cancer	Monotherapy	Preclinical; NA	Cell line	E1	Preclinical migration/invasion; preclinical only	(He et al., 2022)
Downstream	RRM2/HIF-1α	RRM2 or HIF-1α inhibition	ICC	Chemotherapy; monotherapy	Preclinical; NA	Cell line; animal	E1	Preclinical ferroptosis resistance; mechanistic only	(Song et al., 2026)
Downstream	Pyrimidine synthesis	HIF-1α or pyrimidine pathway inhibition	Pancreatic cancer	+ gemcitabine	Preclinical; NA	Cell line; animal	E1	Preclinical gemcitabine resistance; mechanistic only	(Shukla et al., 2017)
Downstream	HPRT1	6-MP (HPRT1 inhibitor)	EGFR-mutant LUAD	+ gefitinib	Preclinical; NA	Cell line; animal	E1	Preclinical DNA damage/apoptosis; mechanistic only	(Geng et al., 2024)
Observational	HIF-1α/LDHA/MCT1/4/GLUT1	NA	Multiple solid tumors	Non-interventional	NA; Observational; NA	Observational human tumor study	E4	Metastasis-free survival; not applicable; prognostic only	(Li et al., 2025b; Hong et al., 2025; Xia et al., 2023; Fallah and Rini, 2019)

+, combined treatment/co-administration with the indicated agent or therapeutic modality. For example, ‘+ bevacizumab’ indicates combination with bevacizumab; ‘+ EGFR, inhibition (gefitinib)’ indicates combination with EGFR, inhibition/gefitinib; ‘+ trastuzumab’ indicates combination with trastuzumab.

**TABLE 4:** Combination strategies, resistance mechanisms, nanomedicine, and biomarkers for HIF-1α-associated metabolic interventions.

Category	Target	Strategy	Combination/context	Resistance	Toxicity	Biomarker	Delivery	Evidence/status	Refs
Combination	MCT1/MCT4	AZD3965 + anti-PD-1	Pancreatic cancer; PD-1/PD-L1-resistant tumors	MCT4 compensation; lactate oxidation; immune heterogeneity	Immune-related AEs	MCT1/HIF-1α co-expression; lactate	NA	Preclinical/early clinical	(Li et al., 2025b; Chen et al., 2022b; Xia et al., 2023)
Combination	PDK1	PDK1 inhibitor (e.g., BX-795) + gefitinib	Glioblastoma	Glycolytic compensation; EGFR bypass; OXPHOS adaptation	EGFR inhibitor toxicity	PDK1/EGFR co-expression; glycolytic flux	NA	Preclinical	(Velpula et al., 2013)
Combination	ACLY/USP13	ACLY/USP13 inhibition + anti-PD-1/PD-L1	Melanoma; HCC	Acetyl-CoA compensation; PD-L1-independent immune evasion; acetate uptake	Immune-related AEs	ACLY/USP13; PD-L1; acetyl-CoA	NA	Preclinical	(Hu et al., 2025; Wang et al., 2024b)
Combination	GLS1	CB-839 + chemotherapy/immunotherapy	CRC; solid tumors	Glutamine synthesis; GDH compensation; glycolysis; mTORC1 rewiring	Not established	GLS1; glutamine flux; GDH	NA	Preclinical/early clinical	(Lv et al., 2025)
Combination	LAT1	JPH203 + mTORC1 inhibition	Glioblastoma; osteosarcoma	BCAT1/BCAT2 compensation; mTORC1 bypass; BCAA recycling	Not established	LAT1; BCAA; mTORC1 activity	NA	Preclinical/early clinical	(Zhang et al., 2021b; Martin et al., 2020)
Combination	Pyrimidine synthesis	HIF-1α/pyrimidine inhibition + gemcitabine	Pancreatic cancer	dCTP salvage; pyrimidine salvage; gemcitabine resistance	Gemcitabine toxicity	dCTP; pyrimidine synthesis enzymes	NA	Preclinical	(Shukla et al., 2017)
Combination	HPRT1	6-MP (HPRT1 inhibitor) + gefitinib	EGFR-mutant LUAD	Purine salvage; HPRT1-independent nucleotide supply; EGFR bypass	Gefitinib toxicity	HPRT1; purine metabolites; EGFR mutation	NA	Preclinical	(Geng et al., 2024)
Resistance	HIF-1α	Direct HIF-1α inhibition	Multiple solid tumors	HIF-2α compensation; spatial/temporal hypoxia heterogeneity; metabolic plasticity	Normal-tissue toxicity; narrow therapeutic window	HIF-1α/HIF-2α; hypoxia gene signature	Hypoxia-responsive nanoparticles; siRNA/CRISPR nanodelivery	Preclinical/early clinical	(Fallah and Rini, 2019)
Resistance	LDHA	LDHA inhibition	Melanoma; HCC; solid tumors	MCT1/MCT4 lactate flux; OXPHOS switch; metabolic plasticity	Not well defined	LDHA; lactate; ferroptosis markers	NA	Preclinical	(Zheng et al., 2024; Yang et al., 2023; Hong et al., 2025)
Resistance	GLUT1	GLUT1 inhibition	Esophageal cancer; LUAD	GLUT3/other transporter compensation; autophagy/ferroptosis escape	Not well defined	GLUT1/EGFR; glucose uptake imaging	NA	Preclinical	(Chen et al., 2022a; Sui et al., 2025; Zhou et al., 2024)
Resistance	MCT1	AZD3965	Lung cancer; HCC	MCT4 compensation; lactate oxidation; immune heterogeneity	Grade 1–2 AEs in Phase I	MCT1/HIF-1α co-expression; lactate	NA	Early clinical	(Li et al., 2025b; Chen et al., 2022b; Xia et al., 2023)
Resistance	SCD1	SCD1 inhibition	Lung cancer; CRC	FADS2 compensation; desaturation reprogramming; AMPK/TGF-β2 survival	Not established	SCD1/FADS2 ratio; membrane lipid composition	NA	Preclinical	(Wang et al., 2026; Zhang et al., 2022)
Resistance	GLS1	GLS1/GDH inhibition	CRC; lung cancer	Glutamine synthesis; GDH compensation; glycolysis; mTORC1 rewiring	Not established	GLS1; glutamine flux; GDH	NA	Preclinical	(Lv et al., 2025; Bian et al., 2023)
Resistance	PHGDH	PHGDH/SHMT2 inhibition	Glioblastoma; CRC	Serine uptake; SHMT2 compensation; one-carbon rewiring	Not established	PHGDH; SAM; serine/glycine flux	NA	Preclinical	(Yun et al., 2023; Zhang et al., 2021a)
Resistance	RRM2	RRM2 inhibition	ICC; breast cancer	dNTP salvage; RRM2-independent replication; ferroptosis escape	Not established	RRM2; dNTP pool; ferroptosis markers	NA	Preclinical	(He et al., 2022; Song et al., 2026)
Resistance	Pyrimidine synthesis	Pyrimidine pathway inhibition	Pancreatic cancer	dCTP salvage; pyrimidine salvage; gemcitabine resistance	Gemcitabine toxicity	dCTP; pyrimidine synthesis enzymes	NA	Preclinical	(Shukla et al., 2017)
Resistance	HPRT1	HPRT1 inhibition	EGFR-mutant LUAD	Purine salvage; HPRT1-independent nucleotide supply; EGFR bypass	Gefitinib toxicity	HPRT1; purine metabolites	NA	Preclinical	(Geng et al., 2024)
Nanomedicine	Mitochondria	PGCE nanoparticles + chemo + anti-angiogenic	Preclinical metastatic models	Poor tumor penetration; hypoxia heterogeneity; off-target toxicity	Off-target toxicity	Mitochondrial targeting	Hypoxia-responsive release	Preclinical	(Sun et al., 2023)
Nanomedicine	HIF-1α	Hypoxia-responsive nanocarriers; siRNA/CRISPR nanodelivery	Multiple solid tumors	HIF-2α compensation; hypoxia heterogeneity	Normal-tissue toxicity	Pharmacodynamic HIF-1α suppression	Hypoxia-responsive; pH-responsive	Preclinical	(Fallah and Rini, 2019; Sun et al., 2023)
Biomarker	Hypoxia/metabolic-immune	Metabolic inhibitor + ICB	Multiple solid tumors	Hypoxia heterogeneity; HIF-2α; immune plasticity	Immune-related AEs	Hypoxia gene signature; HIF-1α/HIF-2α; MCT1; PD-L1; ABCF1-K430la; ctDNA; CTC	NA	Observational/preclinical	(Li et al., 2025b; Hong et al., 2025; Wang et al., 2024b; Xia et al., 2023; Fallah and Rini, 2019)

NA, not applicable or not reported. In the Delivery column, NA, indicates that no specific delivery strategy was described in the cited study, or that a delivery platform is not applicable to the corresponding intervention. In other columns, NA, indicates that the relevant information was not reported or not applicable.

### 3.1 Direct HIF-1α targeting: agents, clinical status, and toxicity

Direct HIF-1α-targeting agents include EZN-2968, PX-478, and HIF-1α siRNA/nanoparticle delivery (Fallah and Rini, 2019) (Table 3). Most are supported by cell-line, animal, or early-phase clinical evidence (mainly E5), and anti-metastatic benefit remains unproven. Because HIF-1α also regulates normal oxygen sensing, glucose homeostasis, vascular adaptation, and cellular stress responses, selectively inhibiting its pro-tumorigenic activity while limiting normal-tissue toxicity remains challenging. CRLX101 is classified as indirect/nanomedicine rather than direct HIF-1α targeting (Table 3).

### 3.2 Downstream metabolic targets: inhibitors, evidence, and resistance

Downstream targets include PDK1, LDHA, GLUT1, MCT1/MCT4, IDH2, FASN, SCD1, CPT1A, ACLY/USP13, GLS1, PHGDH, LAT1, RRM2, pyrimidine synthesis, and HPRT1 (Table 3). These agents may improve tumor selectivity but are vulnerable to metabolic plasticity. Representative compensatory mechanisms include: PDK1 inhibition leading to glycolytic compensation, EGFR bypass, and OXPHOS adaptation; MCT1/MCT4 inhibition leading to MCT4 compensation and lactate oxidation; ACLY/USP13 inhibition leading to acetate/ACSS2-mediated acetyl-CoA compensation; GLS1/GDH inhibition leading to glutamine synthesis, glycolysis, mTORC1 rewiring, and fatty acid oxidation; PHGDH/SHMT2 inhibition leading to serine uptake and one-carbon rewiring; and RRM2/pyrimidine synthesis inhibition leading to dNTP salvage and RRM2-independent replication (Table 4). Notably, agents approved for other indications (e.g., enasidenib for IDH2-mutant AML) are graded E6*, indicating that approval for another indication does not constitute proven anti-metastatic efficacy (Table 3).

### 3.3 Combination strategies and compensatory metabolism

Rational combinations include MCT1/MCT4 inhibition plus anti-PD-1, PDK1 inhibitor plus gefitinib, ACLY/USP13 inhibition plus anti-PD-1/PD-L1, GLS1 inhibition plus chemotherapy or immunotherapy, and LAT1 inhibition plus mTORC1 inhibition (Table 4). Most combination regimens remain at preclinical or early clinical stages (mainly E1–E2 evidence; some reaching E5), and their clinical anti-metastatic efficacy requires prospective validation. Combination design should be guided by target-specific compensatory pathways rather than by single-node inhibition alone.

### 3.4 Nanomedicine and biomarker-guided strategies

Emerging approaches include hypoxia-responsive nanoparticles, mitochondria-targeted delivery, and siRNA/CRISPR nanodelivery (Table 4). Candidate biomarkers include HIF-1α/HIF-2α signatures, hypoxia gene signatures, MCT1/HIF-1α co-expression, lactate, ACLY/USP13, PD-L1, dCTP, and RRM2 (Table 4). Patient stratification by these markers may help identify tumors most likely to benefit from direct HIF-1α inhibition, downstream metabolic blockade, or combined immunometabolic therapy.

Together, Tables 3, 4 provide an updated evidence-graded framework for targeting HIF-1α-associated metabolic vulnerabilities in metastatic cancer.

## 4 Conclusion and perspectives

This review identifies HIF-1α as a central regulator of hypoxia-driven tumor metastasis. By coordinating glucose, lipid, amino acid, nucleotide, and metabolite-associated networks, HIF-1α promotes tumor-cell survival, proliferation, invasion, migration, immune evasion, and distant colonization. These metabolic changes provide energy and biosynthetic precursors while also shaping redox homeostasis and generating signaling metabolites. Therefore, HIF-1α-mediated metabolic reprogramming should be viewed as an integrated tumor-microenvironmental program rather than a set of isolated metabolic alterations.

The effects of HIF-1α extend beyond tumor-cell-intrinsic metabolism. Hypoxia-associated lactate accumulation, extracellular acidosis, altered lipid utilization, nutrient competition, and adenosine signaling reshape interactions among tumor cells, cancer-associated fibroblasts, vascular cells, and immune cells. These changes promote angiogenesis, extracellular-matrix remodeling, immunosuppressive-cell recruitment, and impaired antitumor immunity. Moreover, metabolites can act as signaling molecules and epigenetic regulators that influence tumor plasticity, immune-cell function, and metastatic-niche formation.

Importantly, this program is not uniform across the metastatic cascade. Distinct metabolic modules dominate at different stages, from glycolysis and lactate production during local invasion and circulatory survival to acetyl-CoA, cholesterol, BCAA, and nucleotide synthesis during metastatic outgrowth. Tumor type, metastatic site, and cellular compartment further modify these dependencies.

Several challenges remain. Current studies largely focus on individual HIF-1α-regulated pathways, whereas metabolic crosstalk, compensatory responses, and flux redistribution among glucose, lipid, amino acid, and nucleotide metabolism remain insufficiently defined. Future research should integrate multi-omics profiling, spatial transcriptomics, metabolomics, isotope tracing, and dynamic flux analysis to characterize hypoxic tumors and metastatic niches. Future studies should distinguish HIF-1α downstream, upstream, bypass, and bidirectional feedback mechanisms, as summarized in the regulatory classification table at the end of Section 2. Inhibition of a single metabolic node may redirect flux toward alternative pathways; therefore, compensatory mechanisms should be directly connected to therapeutic targets and validated by isotope tracing and dynamic flux analysis. For example, glycolysis inhibition may redirect flux toward glutaminolysis to maintain energy supply; ACLY inhibition may trigger compensatory acetyl-CoA production from acetate via ACSS2. In addition, HIF-1α functions vary across tumor types, metastatic stages, and cellular compartments. Because HIF-1α also regulates normal oxygen sensing, glucose homeostasis, vascular adaptation, and cellular stress responses, selectively inhibiting its pro-tumorigenic activity while limiting normal-tissue toxicity remains challenging.

Notable limitations persist in current preclinical models. Conventional 2D cell lines lose the metabolic heterogeneity and cell-cell interactions of primary tumors and cannot recapitulate oxygen gradients or the metastatic cascade (Bergers and Fendt, 2021; Faubert et al., 2020). Immunodeficient mouse xenografts lack functional adaptive immunity, preventing evaluation of metabolism-driven immune evasion and immunometabolic combination therapies. Patient-derived organoids preserve the metabolic heterogeneity and cell-cell crosstalk of primary tumors, addressing the key limitations of 2D cell lines (Hu et al., 2025); multiorgan metastasis-on-chip platforms enable precise control of oxygen gradients and recapitulation of the full metastatic cascade, compensating for the shortcomings of immunodeficient mouse models (Zheng et al., 2021). These platforms should be adopted for future mechanistic and therapeutic studies.

Direct HIF-1α targeting faces inherent challenges: HIF-1α mediates physiological oxygen sensing, glucose homeostasis, and vascular adaptation in normal tissues, raising the risk of on-target off-tumor toxicity. In contrast, targeting downstream metabolic dependencies may improve tumor selectivity but is more susceptible to compensatory pathway activation due to metabolic plasticity. This trade-off highlights the need for patient stratification based on metabolic biomarkers.

Intratumoral hypoxia is spatially and temporally heterogeneous, and chronic versus cyclic hypoxia may generate distinct metabolic, vascular, and immune phenotypes. This heterogeneity, together with metabolic plasticity, limits drug penetration and the durability of single-agent HIF-1α inhibition. Therefore, rational combination strategies are needed (Fallah and Rini, 2019), including pairing HIF-1α-targeted therapies with inhibitors of glycolysis, lactate metabolism, lipid synthesis, or nucleotide synthesis, as well as immunometabolic checkpoints, chemotherapy, targeted therapy, anti-angiogenic therapy, or immune checkpoint blockade. These strategies should be stratified by evidence level and integrated with emerging nanomedicine and biomarker-guided approaches, as summarized in Tables 3, 4. Most combination regimens remain at preclinical stage (E2 evidence), and their clinical anti-metastatic efficacy still requires prospective trial validation.

Multi-targeted delivery systems may improve therapeutic efficacy. For example, PGCE nanoparticles co-deliver chemotherapeutic and anti-angiogenic agents and suppress metastasis by targeting tumor-cell mitochondria, illustrating the potential of multi-component strategies to overcome the limitations of conventional HIF-1α inhibition (Sun et al., 2023). Hypoxia-responsive nanocarriers, antibody-based delivery systems, and other tumor-targeted platforms may further improve intratumoral drug accumulation, controlled release, and cell-type selectivity while reducing off-target toxicity.

Although nucleotide-synthesis inhibitors are relatively mature, agents targeting PDK1-related signaling and FASN, including BX-795 and TVB-2640, remain at different preclinical or clinical stages. Metabolic plasticity and inter-/intra-tumoral heterogeneity may enable resistance through compensatory pathway activation. Future efforts should prioritize metabolic subtyping using spatial multi-omics and imaging, biomarker-guided metabolic-immune-targeted combinations, and optimized delivery platforms.

Collectively, the core consensus is that HIF-1α acts as a central regulator integrating glycolytic, lipid, amino acid, and nucleotide metabolic reprogramming to drive all stages of the metastatic cascade. Context-dependent variations exist across tumor types, metastatic sites, and hypoxia modes, leading to heterogeneous therapeutic responses; notably, some pathways such as exogenous citrate exert opposing effects in different glycolytic backgrounds, and certain bypass mechanisms remain debated across studies. Current evidence is strongest for core glycolytic and lactate pathways, whereas many bypass and organ-specific pathways remain understudied. Key unresolved questions include dynamic metabolic crosstalk, clinical efficacy of metabolic targeting, and optimal strategies to overcome hypoxia heterogeneity and compensatory resistance.

In summary, HIF-1α-centered metabolic reprogramming creates multidimensional therapeutic vulnerabilities in tumor metastasis. Targeting these vulnerabilities through spatially informed, biomarker-guided, multimodal interventions may provide new opportunities for clinical management of metastatic cancer.

## Glossary

α-KG: α‐Ketoglutarate
A2AR: Adenosine A2A receptor
ABCF1: ATP-binding cassette subfamily F member
ATF3: Activating transcription factor 3
BCAA: Branched-chain amino acid
BNIP3: BCL2 interacting protein 3
ccRCC: Clear cell renal cell carcinoma
CRC: Colorectal cancer
EGFR: Epidermal growth factor receptor
FFA: Free fatty acid
GBM: Glioblastoma multiforme
H3K27ac: Histone H3 lysine 27 acetylation
H3K9me2: Histone H3 lysine 9 dimethylation
H3K9me3: Histone H3 lysine 9 trimethylation
HIF-2β/ARNT2: Hypoxia-inducible factor 2-beta/ARNT2
JMJD/JMJDs: Jumonji C domain-containing histone demethylase(s)
LUAD: Lung adenocarcinoma
MAPK: Mitogen-activated protein kinase
mTORC1: Mechanistic target of rapamycin complex 1
NADPH: Nicotinamide adenine dinucleotide phosphate
Nrf2/NRF2: Nuclear factor erythroid 2-related factor 2
OXPHOS: Oxidative phosphorylation
PCK1: Phosphoenolpyruvate carboxykinase 1
PFS: Progression-free survival
PUFA: Polyunsaturated fatty acid
SLC1A1: Solute carrier family 1 member 1
TCA: Tricarboxylic acid
TGF-β1: Transforming growth factor-β1
TNBC: Triple-negative breast cancer
USP13: Ubiquitin-specific protease 13
